# Nuclear-Spin Hyperpolarization of Biomolecules by Optically Enhanced NMR at Ultra-High Field

**DOI:** 10.1002/jccs.70254

**Published:** 2026-07-19

**Authors:** Ji Ho Jeong, Anubhab Halder, Silvia Cavagnero

**Affiliations:** Department of Chemistry, University of Wisconsin-Madison, Madison, Wisconsin, USA

**Keywords:** high field, hyperpolarization, LC-photo-CIDNP, NMR, photo-CIDNP, sensitivity

## Abstract

Nuclear magnetic resonance (NMR) is a powerful tool to elucidate molecular structure and dynamics at atomic resolution. Yet, the NMR analysis of biomolecules is often curtailed by low sensitivity and high spectral congestion. High magnetic field strengths (*B*_0_) are in principle capable of overcoming the above limitations. Even at the highest attainable fields, however, unfavorable hardware-, spin-relaxation- and dielectric-related challenges attenuate the attainable sensitivity. Here, we circumvent the above limitations by synergistically combining data collection at 1.1 GHz (25.9 T) with NMR hyperpolarization in liquids. This strategy enables the analysis of aromatic amino acids and proteins at ultra-high sensitivity and resolution. Systematic comparisons between data collected at 1.1 GHz and 600 MHz reveal advantages and disadvantages of both conditions. Hyperpolarization via low-concentration photochemically induced dynamic nuclear polarization (LC-photo-CIDNP) was applied to small-molecule and macromolecular model systems, that is, two tryptophan (Trp) isotopologs and the ^13^C,^15^N-labeled SH3 protein. We show that LC-photo-CIDNP at 1.1 GHz provides both excellent sensitivity (ca. up to 160-fold enhancements relative to non-LC-photo-CIDNP conditions) and remarkable spectral resolution. In all, our results highlight the combination of LC-photo-CIDNP hyperpolarization and ultra-high field as a powerful strategy for the atomic-resolution analysis of biomolecules in solution at low-μM concentration.

## 1 | Introduction

Nuclear magnetic resonance (NMR) spectroscopy is a major technique for the elucidation of molecular structure, dynamics, and interactions at atomic resolution. A key advantage of NMR is its non-invasive nature, rendering it capable of supporting data collection on biological samples under physiologically relevant conditions. Despite its many advantages, however, NMR is constrained by its inherently low sensitivity, which is due to small differences in equilibrium populations of nuclear spin states [1].

In addition, a plethora of biologically important macromolecules, including proteins (especially if highly dynamic and/or very large), nucleic acids, carbohydrates and mixtures of metabolites often display undesirable spectral congestion. The latter arises from insufficient dispersion of NMR resonances across the frequency range pertaining to the nucleus of interest. In turn, spectral congestion and overlaps are often accompanied by ambiguous assignments, and higher-dimensionality [2, 3] or diffusion-based [4] experiments are not always adequate to address the challenge. The above limitations are particularly severe when the molecule of interest experiences both spectral congestion and is highly diluted (e.g., low μM or less) due to either scarce availability, poor solubility, or the need to be handled in dilute form due to high aggregation propensities [5, 6].

A popular strategy to improve both NMR sensitivity and resolution has been to increase the strength of the applied magnetic field (*B*_0_). State-of-the-art commercial superconducting magnets with ^1^H Larmor frequencies exceeding 1 GHz (or 23.5 T) [7, 8] offer substantial gains in spectral resolution due to increased number of Hertz (Hz) per ppm relative to lower-field spectrometers. As far as NMR sensitivity is concerned, this parameter theoretically scales up with *B*_0_^3/2^. In practice, however, sensitivity gains at ultra-high field are highly system- and experiment-dependent [5, 9, 10]. For instance, in the case of globular proteins, sensitivity gains at very high field (e.g., 1.1 GHz) are often minimal, relative to more conventional fields (e.g., 600 MHz). In contrast, unfolded and(or) highly dynamic proteins, including intrinsically disordered proteins (a.k.a., IDPs, e.g., α-synuclein), tend to experience higher sensitivity benefits at ultra-high field [9, 10]. The latter outcome likely originates from favorable field-dependent transverse relaxation [9, 10]. Namely, relaxation rates of globular proteins are dominated by dipole–dipole interactions and chemical shift anisotropy (CSA), with the weight of the CSA contribution scaling up with $B_{0}^{2}$ [11–13]. At ultra-high field, the above effects accelerate the transverse relaxation rate constants (*R*_2_) of globular proteins, resulting in extensive line-broadening and decreased resonance intensities. In the case of dynamic biomolecules and IDPs, which are characterized by faster and less anisotropic local and global motions than globular proteins, the above unfavorable effects tend to be more moderate. Chemical exchange phenomena also play a role in the observed nuclear relaxation rates [14], and favor or disfavor the signal-to-noise ratio (S/N) of highly dynamic systems at ultra-high field depending on their conformational-exchange rate constants [9, 14].

**FIGURE 1 |:**
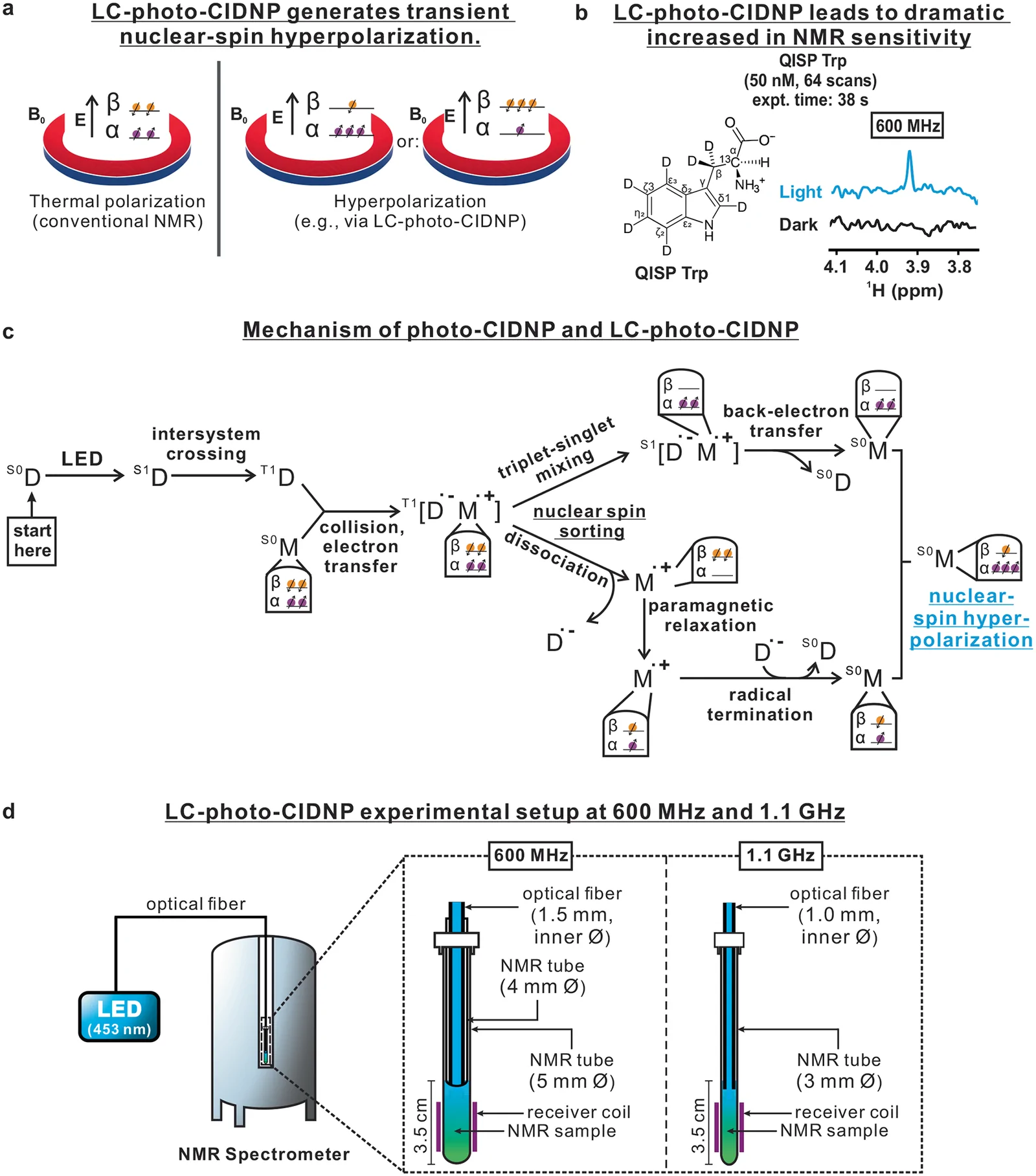
Overview of low-concentration photochemically induced dynamic Nuclear Polarization (LC-photo-CIDNP) NMR and experimental set-up at 600 MHz and 1.1 GHz. (a) Transient nuclear-spin hyperpolarization generated by LC-photo-CIDNP NMR. (b) One-dimensional LC-photo-CIDNP (^1^H^α^ region) spectrum of a 50 nM quasi-isolated-spin-pair (QISP) Trp sample under light (LED-on) and dark (LED-off) conditions. Data were collected at 25°C on a 600 MHz spectrometer equipped with a cryogenic probe from Bruker (cryoprobe). The sweep width was 10,000 Hz, and 64 scans were acquired with 4096 total points. The recycle delay and LED irradiation time were 0.05 s and 0.2 s per scan, respectively. The sample includes 2.5 μM fluorescein photosensitizer (see Section 3 for details). (c) Schematic representation of photo-CIDNP and LC-photo-CIDNP mechanisms. (d) LED-assisted LC-photo-CIDNP experimental setup employed at 600 MHz and 1.1 GHz NMR spectrometers. A 3-mm and a 5-mm (outer) diameter NMR tube were used at 1.1 GHz and 600 MHz, respectively. At 600 MHz only, a 4-mm-diameter NMR tube was used as a glass insert hosting the end of the optical fiber devoted to sample irradiation (see Section 2 and Section 3 for more details).

While originally mostly employed to probe surface accessibility of macromolecules [15–18], photochemically induced dynamic nuclear polarization (photo-CIDNP) has recently emerged as a powerful strategy to increase NMR sensitivity [5, 19–22]. This technology benefits from depletion of molecular oxygen in solution to retard photodegradation of dye and molecule of interest [23], and takes advantage of careful assessment of key parameters governing various aspects of the mechanism [20]. Photo-CIDNP generates significant nuclear-spin hyperpolarization that enhances the signal-to-noise (S/N) of aromatic amino acids, either as free species or as solvent-accessible residues within proteins, as well as neurotransmitters and pharmaceuticals in either aqueous buffer or crowded cellular matrices [19, 24–26]. In contrast to other hyperpolarization approaches applicable to liquids [19], photo-CIDNP is extremely biocompatible, experimentally accessible, and can be readily implemented on any NMR spectrometer at low cost [19]. Recent methodological improvements, including photosensitizers with long triplet-state lifetimes, improved pulse sequences, mitigation of singlet-oxygen-mediated degradation, and selective isotopic labeling, pushed the concentration limit of photo-CIDNP down to the low-nanomolar regime and extended the number of sensitivity-enhanced resonances [25–28]. These advances are recapitulated by the acronym low-concentration photo-CIDNP, or LC-photo-CIDNP, which has been used to denote this approach [16, 23, 28–31]. Figure 1a provides a simple conceptual illustration of the transient hyperpolarization principle. A representative LC-photo-CIDNP spectrum of a 50 nM sample of the amino acid tryptophan (Trp) is shown in Figure 1b. Further, LC-photo-CIDNP can be easily extended to multidimensional NMR, enabling ^1^H-^13^C- and ^1^H-^15^N correlation experiments on amino acids, peptides, and proteins at low-micromolar and sub-micromolar levels within only a few minutes [15, 27, 32–34].

Importantly, the field dependence of LC-photo-CIDNP is fundamentally distinct from that of conventional thermal polarization, and it exhibits a complex nonlinear profile [35]. A scheme highlighting the major mechanistic steps underlying photo-CIDNP and LC-photo-CIDNP [20–22, 36–39] is shown in Figure 1c. After formation of a ^T1^[D^.−^M^.+^] triplet radical pair, nuclear spin sorting gives rise to geminate recombination of nuclear-spin states that undergo fast (ca. 100 ns) triplet-singlet (TS) mixing. Conversely, nuclear-spin states characterized by slow (>> 100 ns) TS mixing undergo triplet radical-pair dissociation, followed by paramagnetic relaxation and radical termination. The latter process also generates additional enhancements known as F-pair polarization [22, 36].

Previous computations, based exclusively on geminate-polarization arguments [35, 40, 41], predicted that the polarization of uniformly labeled ^13^C,^15^N tryptophan (Trp-U-^13^C,^15^N) and Trp isotopologs bearing a quasi-isolated spin-pair (QISP Trp) does not differ substantially at 14.1 T (600 MHz) and 25.9 T (1.1 GHz) [41, 42]. On the other hand, experimentally observed steady-state LC-photo-CIDNP arises from both fast (ca. 100 ns) geminate and slow (ca. 100 μs) F-pair polarization. In addition, longitudinal relaxation (*T*_1_) of the nuclei of interest also plays a role in the extent of the observed hyperpolarization [20, 22]. Moreover, at ultra-high field, additional factors including probe geometry, radio-frequency coil heating and variations in optical excitation efficiency may further complicate the observed LC-photo-CIDNP response. Thus, the field dependence of LC-photo-CIDNP at ultra-high field remains poorly understood. In addition, to date no systematic experimental investigations combining ultra-high-field data collection with LC-photo-CIDNP hyperpolarization have been carried out. Therefore, the feasibility of synergistic dramatic sensitivity enhancements arising from both ultra-high-field and LC-photo-CIDNP remains unexplored.

Here, we address the above gap of knowledge by performing the first implementation of LC-photo-CIDNP on an ultra-high-field 1.1 GHz (25.9 T) NMR spectrometer. Our overall aim is to explore the potential for a combined highly sensitive and high-resolution analysis of biomolecules, especially in cases where very-high-field alone does not lead to large enhancements. We provide systematic comparisons between spectroscopic performances at 600 MHz and 1.1 GHz, focusing on experimental sensitivity and spectral resolution at low-micromolar concentration. We employ one- and two-dimensional ^1^H–^13^C correlation experiments, that is, ^1^H-detected ^13^C photo-CIDNP (a.k.a. ^13^C RASPRINT). In addition, we present data on two selectively labeled Trp isotopologs, as well as the uniformly ^13^C,^15^N-labeled N-terminal SH3 domain of the *Drosophila melanogaster* adaptor protein drkN (PDB: 2A36, denoted here as SH3) in aqueous buffer. The slow exchange (on the chemical-shift timescale) between the SH3 folded and unfolded states, coupled with the comparable population of these states, enable investigating the effect of ultra-high-field LC-photo-CIDNP on both the native and unfolded conformations. These features render SH3 an excellent model system for concurrently investigating the effect of ultra-high-field LC-photo-CIDNP hyperpolarization on conformationally disordered and structured protein states. For both free amino acids and the SH3 protein, the experimentally observed LC-photo-CIDNP enhancements are overall consistent with theoretical expectations. Importantly, the data demonstrates that LC-photo-CIDNP at 1.1 GHz provides > 100-fold resonance-area enhancements relative to conventional NMR at this field, along with excellent gains in spectral resolution. In addition, this work provides conceptual and practical frameworks for understanding the tradeoffs that govern variations in sensitivity and resolution when LC-photo-CIDNP is carried out at ultra-high field.

## 2 | Results and Discussion

### 2.1 | Mechanism and Practical Implementation of Ultra-High Field LC-Photo-CIDNP

At its core, improving NMR sensitivity involves transiently altering the populations of nuclear spin states. Spin hyperpolarization methodologies including parahydrogen-induced polarization (PHIP), dynamic nuclear polarization (DNP), Overhauser DNP (oDNP), dissolution DNP (dDNP), and LC-photo-CIDNP [19, 43], achieve this goal by driving nuclear and/or electron spins away from thermal equilibrium, thereby producing non-equilibrium states with large transient population differences that eventually lead to enhanced NMR signal-to-noise (S/N) ratios [5, 19, 44]. In some cases, the observed enhancements are truly dramatic. Therefore, we explored the effect of combining an ultra-high *B*_0_ (1.1 GHz ^1^H Larmor frequency) with LC-photo-CIDNP non-equilibrium hyperpolarization in liquids. We reasoned that in principle, this strategy may afford unprecedented NMR sensitivity and resolution.

Similarly to photo-CIDNP, the mechanism of LC-photo-CIDNP in liquids proceeds via a transient triplet-state spin-correlated radical pair, which gets generated upon diffusional encounter between the molecule of interest and a photoexcited triplet dye followed by electron transfer (Figure 1c) [45, 46]. Photosensitizer dyes with long triplet-state lifetimes increase the probability of radical-pair formation in liquids [29, 47]. Subsequent nuclear-spin-dependent TS interconversion within the radical pair leads to spin-selective recombination and escape pathways leading to spin sorting, as schematically illustrated in Figure 1c, for spin-1/2 nuclei [20]. The recombination path leads to geminate polarization, which is generated within tens to hundreds of nanoseconds. The escape path is mostly adopted by nuclear spin states with slower TS mixing rates. In this case, the radical-pair components diffuse apart within hundreds of microseconds, subsequently relaxing back to thermal equilibrium via paramagnetic relaxation followed by radical encounter, some statistically controlled F-pair nuclear polarization, and back-electron transfer. The observed steady-state LC-photo-CIDNP enhancement ultimately arises from the combined contributions of geminate and escape polarization paths (Figure 1c).

When implementing LC-photo-CIDNP experiments on an ultra-high-field spectrometer (i.e., 25.9 T, corresponding to 1.1 GHz ^1^H Larmor frequency), several practical hardware-related considerations needed to be taken into account.

First, due to extensive dielectric-constant-related line-broadening and due to limited RF power tolerance at 1.1 GHz, the receiver coil’s diameter is often reduced to enable uniform excitation at the available radio-frequency (RF) power [1]. As a consequence, experiments at 1.1 GHz have been performed on a 3-mm probe in a 3-mm outer-diameter NMR tube (Figure 1d), resulting in a smaller sample volume (within the RF detection region) than corresponding experiments carried out in the presence of a 5-mm cryogenic probe at 600 MHz [9, 48, 49].

Second, the reduced sample-tube diameter at 1.1 GHz demands the use of a different fiber-optic diameter and overall setup, as illustrated in Figure 1d. Namely, an optical fiber with an inner diameter of 1.0 mm had to be used, as opposed to the 1.5 mm optical fiber typically employed for LC-photo-CIDNP experiments carried out in 5-mm NMR sample tubes. In addition, given the narrower NMR tube diameter that had to be used at 1.1 GHz, instead of employing an optical fiber enclosed within a glass coaxial-insert sleeve, the fiber had to be introduced directly into the NMR tube.

Third, the much taller 1.1 GHz magnet demanded a longer optical-fiber length (10 m) at 1.1 GHz than at 600 MHz (4.1 m). Despite the above fiber cross-sectional area and fiber-length differences, the LED powers at the fiber tips were adjusted to be very similar in the 600 MHz and 1.1 GHz experiments (see Section 3).

In summary, the practical setup differences at 1.1 GHz relative to 600 MHz include a smaller-diameter probe and sample tube, and thinner fiber optic, potentially leading to variable axial-illumination profiles (Figure 1d). On the other hand, the multiple LED-light reflections within the sample tube, described in detail in the supporting material of the work by Yang et al. [24], render optical illumination uniform, including across vertical and transverse planes. This effect is expected to be operative across NMR sample tubes at both applied fields. Yet, the effective number of detected molecules of interest is smaller at 1.1 GHz than at 600 MHz, due to the smaller detected observation volume. This effect clearly decreases the sensitivity advantage of ultra-high-field spectrometers, further motivating the implementation of high-field hyperpolarization.

### 2.2 | Nuclear-Spin Hyperpolarization at 1.1 GHz Leads to Large NMR Sensitivity Enhancements

**FIGURE 2 |:**
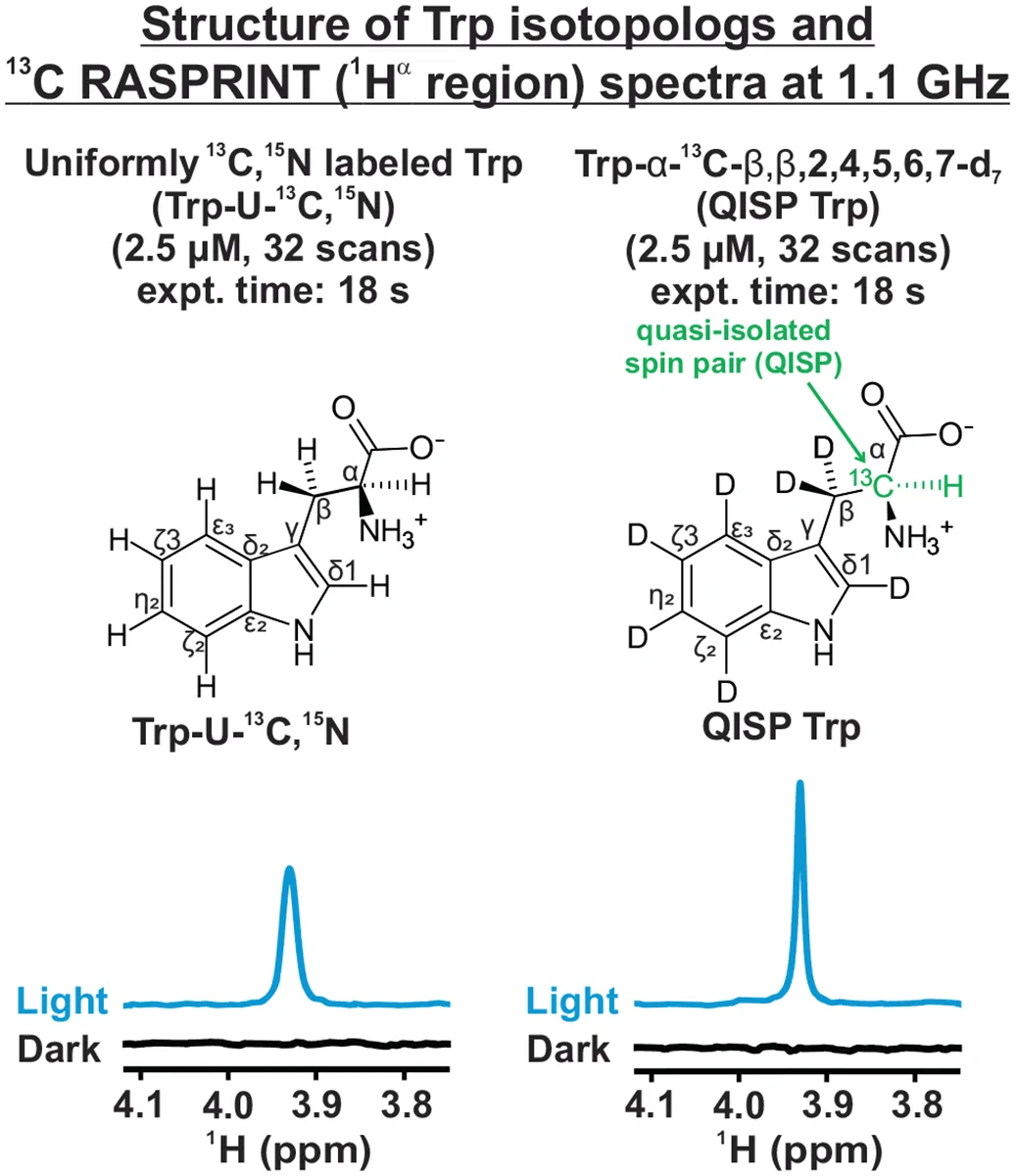
One-dimensional ^13^C RASPRINT (^1^H^α^ region) NMR spectra of Trp-U-^13^C, ^15^N and QISP Trp display greatly enhanced sensitivity at 1.1 GHz. ^13^C RASPRINT data were collected at 2.5 *μ*M sample concentration at 25°C with a 12,500 Hz ^1^H sweep width, 4096 total points and 32 scans. LED irradiation and recycle delay were set to 0.2 s and 0.05 s respectively. Both samples contained 5 *μ*M fluorescein photosensitizer. The 1.1 GHz spectrometer was equipped with a cryogenic probe (see Section 3 for additional details).

The chemical structure and the ^13^C RASPRINT LC-photo-CIDNP spectra of two tryptophan (Trp) isotopologs, for data collected at 25.9 T (1.1 GHz), are shown in Figure 2. The ^13^C RASPRINT experiment capitalizes on ^13^C photo-CIDNP, which is detected via ^1^H NMR after ^13^C-to-^1^H coherence transfer via reverse-INEPT. The two compounds that were independently analyzed are uniformly ^13^C,^15^N labeled tryptophan (Trp-U-^13^C,^15^N) and a selectively labeled Trp isotopolog bearing a quasi-isolated spin pair (QISP) [25]. The latter species is denoted as QISP Trp, and it bears 7 hydrogen-to-deuterium replacements (Trp-α-^13^C-β,β,2,4,5,6,7-d_7_). The QISP labeling strategy leads to particularly large sensitivity enhancements due to both dark and light effects, which are optical-irradiation independent and dependent, respectively, as described [25].

The dark effects arise from the simplified nuclear-spin network, leading to longer nuclear transverse relaxation times (T_2_s), and from the highly reduced number of ^1^H-^1^H, ^13^C-^13^C and ^1^H-^13^C scalar couplings, which decrease magnetization losses during the pulse sequence. The light effects arise from the reduced contribution of hyperfine-coupling-constant terms (A_i_) to the overall rate of LC-photo-CIDNP geminate recombination. The latter is due to (a) the smaller A_i_ values of deuterated nuclei of QISP amino acids relative to their protonated isotopologs, and (b) the effective lack of hyperfine couplings between unpaired electrons within the radical pair and natural-abundance, that is, non-^13^C-enriched, carbon nuclei [25]. The preserved ^13^C^α^-^1^H^α^ pair of QISP amino acids enables selective detection of these two diagnostic nuclei (^13^C^α^ and ^1^H^α^) in either ^1^H-detected or ^13^C-detected experiments.

**FIGURE 3 |:**
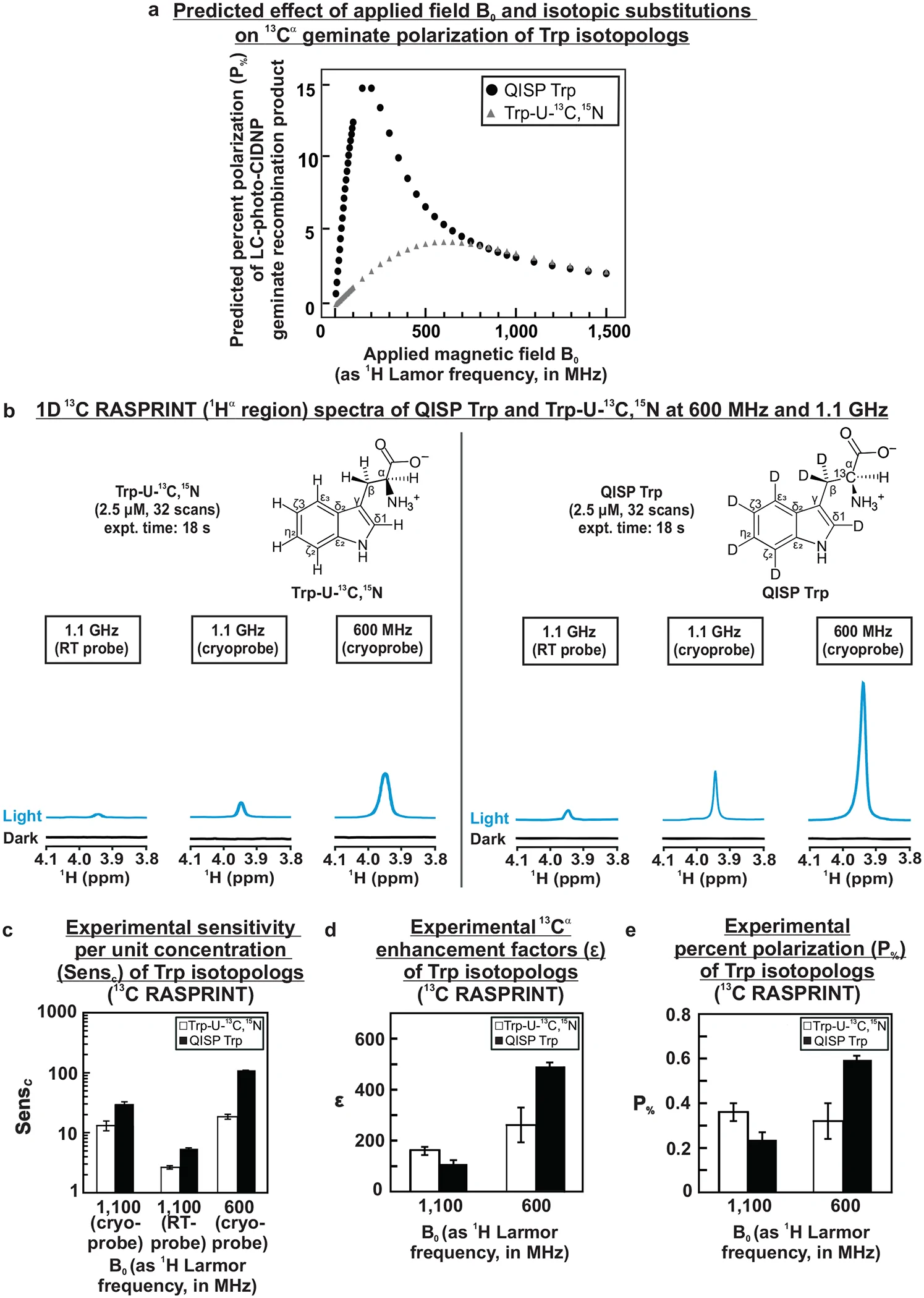
LC-photo-CIDNP nuclear-spin hyperpolarization leads to dramatic enhancements, polarizations and sensitivities at both 600 MHz and 1.1 GHz. (a) Prediction of field-dependent ^13^C^α^ LC-photo-CIDNP geminate polarization of Trp-isotopologs illustrates the expected trends. QISP Trp (black circles) exhibits strong field-dependent variations in geminate polarization, with a maximum at low field, while Trp-U-^13^C, ^15^N (gray triangles) shows a weaker field dependence. Data were modified with permission from S. Y. Li et al. *Appl. Magn. Reson*. 2023, 54, 59–75. (b) One-dimensional ^13^C RASPRINT (^1^H^α^ region) spectra of QISP Trp and Trp-U-^13^C, ^15^N were acquired at 600 MHz with a cryoprobe from Bruker, and at 1.1 GHz with either a cryoprobe or a room-temperature (RT) probe. For both isotopologs, data were collected at 25°C under light (0.2 s LED irradiation, 0.05 s recycle delay) and dark (LED-off) conditions at 2.5 *μ*M sample concentration with a sweep width of 10,000 Hz (600 MHz) or 12,500 Hz (1.1 GHz), 4096 total points, and 32 scans. All samples contained 5 μM fluorescein photosensitizer (see Section 3 for details). (c) Sensitivity per unit concentration (Sens_*c*_, note the y axis is on a log_10_ scale) deduced from ^13^C RASPRINT (^1^H^α^ region) experiments under light conditions, on a 600 MHz (with a cryoprobe) and 1.1 GHz (with a cryoprobe or a RT probe as shown in the figure) spectrometer, (d) experimentally determined ^13^C^α^ enhancement factors (ε) and (e) percent polarizations (P_%_) of the two Trp isotopologs determined upon ^13^C RASPRINT (^1^H^α^ region) data collection under light conditions, at 600 MHz with a cryoprobe (*n* = 2) and at 1.1 GHz NMR with a RT probe (*n* = 2). Note that ε values (computed according to Equation (2)) and P_%_ values (computed from ε according to Equation (3)) are expected to be independent of probe type, given that both light and dark spectra at each field were acquired with the same probe. Error bars in panels (c–e) denote standard deviations from the mean.

Figure 3a displays the computationally predicted dependence of nuclear-spin hyperpolarization on *B*_0_ for the same two Trp isotopologs analyzed in Figure 2. We employed theoretical expressions for the ^13^C^α^ photo-CIDNP geminate recombination rate constant and for the overall ^13^C hyperpolarization according to Adrian as described [42, 50]. The predicted ^13^C^α^ polarization of U-^13^C, ^15^N Trp exhibits a relatively weak field dependence, reaching a maximum at ca. 600 MHz. In contrast, QISP Trp displays a much more pronounced field dependence, with a significantly higher polarization maximum at ca. 200 MHz. The difference in LC-photo-CIDNP polarization between 600 MHz and 1.1 GHz can be understood in terms of the *B*_0_ dependence of the TS mixing frequency within the photo-CIDNP radical pair [42]. Despite the fact that the overall ^13^C geminate polarization of both Trp isotopologs at 1.1 GHz is predicted to be smaller than the corresponding value at 600 MHz, a significant extent (ca. 3%) of percent geminate-polarization enhancement (P_%_) is predicted at 1.1 GHz, rendering the experiment highly desirable for sensitivity enhancement purposes. This result is qualitatively consistent with the experimental spectra acquired at 1.1 GHz, shown in Figure 2.

In summary, given the extent of experimentally verified and predicted hyperpolarization at 1.1 GHz, the data in Figures 2 and 3a show that LC-photo-CIDNP at ultra-high field has a remarkable capability to achieve dramatic NMR sensitivity enhancements. The significance of this result is further testified by the fact that the dark spectra of both isotopologs show no signal at all, under dark conditions at 1.1 GHz (Figure 2). Additional quantitative assessments in support of this concept are provided in the next section.

Incidentally, the ca. 2-fold greater resonance intensity of QISP Trp relative to the Trp-U-^13^C, ^15^N uniformly ^13^C,^15^N-labeled isotopolog in Figure 2 is not accounted for by the theoretical predictions of Figure 3a. This outcome can be explained on the grounds of the previously identified dark effects, which require no LED irradiation and yet contribute to the observed LC-photo-CIDNP enhancements [25]. These dark effects are not included in the geminate-polarization-based predictions of Figure 3a and include longer T_2_s (decreasing linewidths) and a simplified scalar-coupling network (increasing areas) for QISP Trp relative to Trp-U-^13^C, ^15^N during the ^13^C RASPRINT pulse sequence [25].

### 2.3 | LC-Photo-CIDNP Sensitivities, Enhancement Factors and Polarizations at 1.1 GHz and 600 MHz of Trp Isotopologs Are in Remarkable Agreement With Theoretical Predictions

^13^C RASPRINT (^1^H^α^ region) spectra of QISP Trp and U-^13^C, ^15^N Trp, for data collected at both 1.1 GHz (with a room-temperature (RT) probe and a cryoprobe from Bruker) and 600 MHz (with a cryogenic probe from Bruker (cryoprobe)), are shown in Figure 3b. In both cases, spectra were acquired ultra-rapidly (18 s) under light (LED-on) conditions at 2.5 μM sample concentration with 32 scans. Reference dark spectra collected under LED-off conditions are also shown. Interestingly, QISP Trp shows substantially stronger resonance intensities at 600 MHz than at 1.1 GHz (right panel, Figure 3b), with no discernible signals detected under dark conditions at both fields. On the other hand, Trp-U-^13^C,^15^N shows minor differences in LED-on resonance intensities at the two fields (left panel, Figure 3b).

For both Trp isotopologs, we quantitatively evaluated NMR sensitivity of per-unit-concentration (Sens_*c*_) according to relation

(1) $${\text{Sens}}_{c}=\frac{\left(\frac{s}{N}\right)}{[C]*\surd t},$$

where [*C*] and *t* denote the concentration of the molecule of interest and the total experimental time, respectively [26]. The Sens_*c*_ values for QISP Trp and Trp-U-^13^C,^15^N at 600 MHz and 1.1 GHz NMR are shown in Figure 3c and Table 1. QISP Trp shows considerable Sens_*c*_ enhancement at both 600 MHz (~5.9 fold) and 1.1 GHz (~2.0-fold), relative to Trp-U-^13^C,^15^N. This difference likely arises from a combination of light- and dark-state effects [25, 26]. We previously showed that the selectively labeled QISP Trp enhances overall sensitivity even in the absence of optical irradiation [25, 26], relative to Trp-U-^13^C,^15^N. For instance, sharper linewidths arising from longer T_2_ relaxation times, together with reduced signal loss due to the elimination of the extensive J-coupling network, were shown to contribute to the sensitivity enhancement [25, 26].

To better understand how the light and dark effects contribute to Sens_*c*_ differences between the two Trp isotopologs, we evaluated enhancement factors (ε) and percent polarization (P_%_) values, as shown in Figure 3d,e. These parameters are unique reporters of the LC-photo-CIDNP light effect [25, 26]. The enhancement factor ε reflects the resonance-area enhancement under light conditions (relative to the corresponding dark signal) and was calculated according to

(2) $$\varepsilon =\frac{{\text{Area}}_{\text{light}}{\left[Trp\right]}_{\text{dark}}}{{\text{Area}}_{\text{dark}}{\left[Trp\right]}_{\text{light}}},$$

where area and [Trp] denote the ^1^H^α^ resonance area and Trp concentration, respectively, under light (LED-on) and dark (LED-off) conditions. The experimental ε values for QISP Trp and Trp-U-^13^C,^15^N at both fields are plotted in Figure 3d and numerically shown in Table 1. These data demonstrate a strong *B*_0_ dependence for both QISP Trp and Trp-U-^13^C,^15^N. At 600 MHz, QISP Trp exhibits significantly larger (*p* = 0.03, Student’s 2-tailed *t*-test) enhancements (ε = 487.6) than Trp-U-^13^C,^15^N (ε = 261.2). In contrast, at 1.1 GHz, both Trp isotopologs show substantial enhancements (ε = 104.8 for QISP Trp and ε = 160.1 for Trp-U-^13^C,^15^N), but the difference between them is no longer statistically significant (*p* = 0.15). The ^13^C^α^ percent polarization (P_%_) was then determined via Equation (3) below, which accounts for Boltzmann populations at the respective fields and ε values

(3) $${\text{P}}_{\%}={\varepsilon \text{P}}_{\%,\text{te}},$$

where *P*_%,*te*_ is the percent polarization at thermal equilibrium according to

(4) $${\text{P}}_{\%,\text{te}}=\text{tanh}\left(\frac{\hbar \gamma {\text{B}}_{0}}{2{\text{k}}_{\text{B}}\text{T}}\right)\times 100,$$

where *γ* is the gyromagnetic ratio (with $\gamma_{13_{C}}=10.705\;\text{MHz}/T,\;\gamma_{1_{H}}=42.576\;\text{MHz/T}$) [47], *B*_0_ is the applied *B*_0_ in Tesla, and T is the sample temperature in K. The results are shown in Figure 3e and assessed in Table 1. The trends in the ε and P_%_ values are in remarkable agreement with the theoretical predictions of geminate polarization in Figure 3a. Namely, QISP Trp exhibits better performance than Trp-U-^13^C,^15^N at 600 MHz (*p* = 0.02), while the two isotopologs display identical polarizations at 1.1 GHz within error (*p* = 0.07). It is worth noting that the nuclear ^13^C^α^ T_1_ is a contributor to LC-photo-CIDNP under steady-state conditions [20, 27]. In our case, identical ^13^C^α^ T_1_ values within errors were observed (Table S1) for both Trp isotopologs at 600 MHz and 1.1 GHz. This result rules out nuclear ^13^C^α^ T_1_ as a contributing factor to the observed ^13^C RASPRINT spectral data.

In order to quantify the first type of dark effects arising from the narrower linewidths of QISP Trp relative to Trp-U-^13^C,^15^N [25, 26], we measured ^1^H^α^ T_2_ relaxation times of both Trp isotopologs at both fields. The values are summarized in Table S2. QISP Trp shows longer ^1^H^α^ *T*_2_ values at both fields, relative to Trp-U-^13^C,^15^N. This result is consistent with narrower ^1^H linewidths. On the other hand, *T*_2_ relaxation for Trp isotopologs at 600 MHz and 1.1 GHz is the same within error (*p* = 0.34 for QISP Trp and *p* = 0.68 for Trp-U-^13^C,^15^N). Note that any field inhomogeneity contributions to linewidth are not accounted for in these *T*_2_ measurements. The second type of dark effects arises from reduced coherence loss during the pulse sequence due to removal of the scalar coupling network (^13^C–^13^C, ^1^H–^13^C, and ^1^H–^1^H) in QISP Trp relative to Trp-U-^13^C,^15^N [25]. This effect was quantified by comparing the ^1^H^α^ resonance area per unit concentration under dark conditions. As shown in Table 2, QISP Trp exhibits a ~200% increase in resonance area at 1.1 GHz, compared to a ~42% increase at 600 MHz, relative to Trp-U-^13^C,^15^N.

All the above light and dark effects independently contribute to the overall observed sensitivity (Sens_*C*_) and are quantitatively reported at both 600 MHz and 1.1 GHz in Tables 2 and 3, respectively. For added clarity, all values are shown relative to Trp-U-^13^C,^15^N. Notably, the product of the three individual contributions (one light and two dark effects) reproduces the overall experimentally observed Sens_*C*_ values at 600 MHz (*p* = 0.32, Table 2) and 1.1 GHz (p = 0.14, Table 3). These results provide excellent support for our predicted origin of the observed effects [25, 26].

In summary, QISP Trp exhibits substantially greater enhancement factors, percent polarization, and overall NMR sensitivity than Trp-U-^13^C,^15^N at 1.1 GHz. These results demonstrate that increasing the applied *B*_0_ does not necessarily lead to improved LC-photo-CIDNP sensitivity in uniformly labeled systems. Although the enhancement at 1.1 GHz is not as high as at 600 MHz, it is important to note that significant hyperpolarization is still effectively achieved, demonstrating the robust performance of the LC-photo-CIDNP with QISP Trp even at ultra-high fields.

### 2.4 | LC-Photo-CIDNP Hyperpolarization at 1.1 GHz Provides Dramatically Enhanced Sensitivity and Resolution for the Structural Analysis of Proteins

**FIGURE 4 |:**
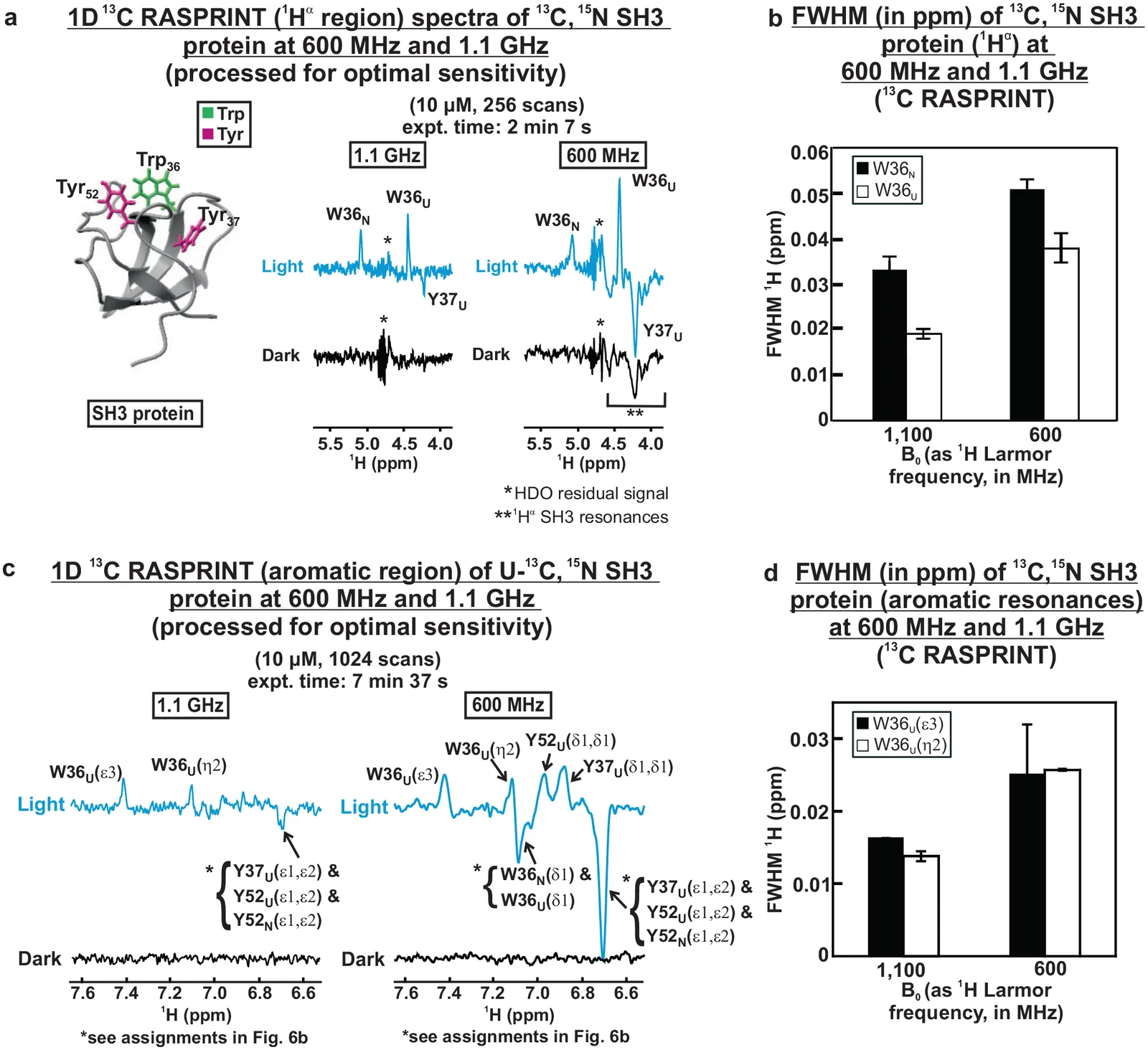
^13^C RASPRINT (^1^H^α^ region) detection of ^13^C,^15^N-labeled SH3 protein at 600 MHz and 1.1 GHz. (a) Structure of SH3 protein and one-dimensional ^13^C RASPRINT (^1^H^α^ region) spectra of the SH3 protein acquired under light (LED-on) and dark (LED-off) conditions. Spectra were collected at 10 *μ*M SH3 concentration at 25°C with a sweep width of 12,500 Hz (1.1 GHz) or 10,000 Hz (600 MHz), 4096 total points and 256 scans. A cluster of ^1^H^α^–^13^C^α^ resonances from the SH3 protein appears around 4.2 ppm in the dark spectrum (data not shown). The constant time evolution (T) for ^13^C RASPRINT (^1^H^α^ region) was set to 26.6 ms (1/*J*_*CαCβ*_). (b) FWHM values (in ppm) of selected ^1^H^α^ resonances (e.g., W36_U_ and W36_N_) deduced from ^13^C RASPRINT (^1^H^α^ region) spectra at 600 MHz and 1.1 GHz (*n* = 2, for each field). (c) One-dimensional ^13^C RASPRINT (aromatic region) spectra of the SH3 protein acquired under light (LED-on) and dark (LED-off) conditions. Spectra were acquired at 10 *μ*M SH3 concentration at 25°C with a sweep width of 16,666 Hz (1.1 GHz) or 10,000 Hz (600 MHz), 4096 total points and 1024 scans. The constant-time evolution (T) for ^13^C RASPRINT (aromatic region) was set to 26.6 ms (2JCCaromatic). (d) FWHM values (in ppm) of selected aromatic resonances (e.g., W36_U_ (ε3) and W36_U_ (*η*2)) obtained from ^13^C RASPRINT (aromatic region) spectra at 600 MHz and 1.1 GHz (*n* = 2, for each field). The LED irradiation time per scan and recycle delay were 0.2 s and 0.05 s, respectively. Samples contained the fluorescein photosensitizer at 8 *μ*M concentration (see Section 3 for details). All data shown in this figure were collected on spectrometers equipped with a cryogenic probe from Bruker (cryoprobe). Error bars in panel b and d denote standard deviations from the mean.

The N-terminal domain of the *Drosophila melanogaster* adaptor protein drkN, denoted here as SH3, was employed as a model system to investigate LC-photo-CIDNP at ultra-high *B*_0_. The slow chemical exchange (on the NMR chemical-shift timescale) for the interconversion between SH3 native and unfolded conformations enables simultaneous observation of both conformational states [51]. The folded-to-unfolded population ratio is ca. 3:2 at room temperature [52]. The SH3 native-state 3D structure in Figure 4a highlights the single Trp and two Tyr residues (W36, Y37 and Y52) of this protein, which are all solvent-exposed and LC-photo-CIDNP active [24, 27, 28]. Panels a and c of Figure 4 show the LC-photo-CIDNP spectra of the uniformly ^13^C, ^15^N-labeled SH3 protein (10 μM) at 600 MHz and 1.1 GHz. In addition, panels b and d of Figure 4 provide linewidths (in ppm) of ^1^H^α^ and aromatic-proton resonances via the frequency-width-at-half-maximum (FWHM) parameter. ^1^H-detected 1D ^13^C RASPRINT data on the aliphatic and aromatic regions of SH3 were collected separately at 600 MHz and 1.1 GHz at 10 μM protein concentration, to assess *B*_0_-dependent changes in sensitivity and spectral resolution. All spectra were acquired under both light and dark conditions. As shown in Figure 4a, distinct resonances corresponding to unfolded (U) and native (N) Trp residues that is, W36_U_ and W36_N_, are observed under light conditions [52], while no detectable resonances are present in the corresponding dark spectra. Both field strengths enable ready detection of the target resonances under light conditions. On the other hand, the 1.1 GHz spectrum reproducibly exhibits sharper lineshapes due to the increased spectral resolution (in ppm) at ultra-high field.

It is worth noting that the FWHM values of native-state Trp (W36_N_) in Hz actually increase from 30.5 ± 1.3 Hz at 600 MHz to 36.5 ± 3.4 Hz at 1.1 GHz, while the FWHM values of unfolded Trp (W36_U_) in Hz remain nearly unchanged from 22.8 ± 1.9 at 600 MHz to 21.2 ± 1.0 Hz at 1.1 GHz (Figure S1). This observation is consistent with the expected larger contribution of CSA to nuclear-spin relaxation at higher *B*_0_ (governed by a *B*_0_^2^ dependence) [53]. For the SH3 native state, molecular motions are dominated by the overall rotational correlation time (*τ*_C_). Thus, the higher CSA relaxation-rate contribution at higher field, which is directly proportional to *τ*_C_, results in additional line broadening. In contrast, the highly dynamic unfolded state undergoes fast local motions, which decrease the global effective *τ*_C_ and cause weaker CSA contributions to transverse relaxation, thereby producing nearly field-independent linewidths. However, when expressed in ppm, FWHM values are normalized according to the spectrometer frequency. As a consequence, despite the modest increase in linewidths in Hz at the higher *B*_0_, linewidths in ppm end up being narrower, resulting in an overall improved spectral resolution. Figure 4b shows a quantitative comparison between FWHM values (in ppm) for the W36_U_ and W36_N_^1^H^α^ resonances at both fields. The FWHM values of the W36_U_ and W36_N_ resonances at 600 MHz are 0.051 ± 0.002 ppm and 0.038 ± 0.003 ppm, respectively. At 1.1 GHz, FWHM values decrease to 0.033 ± 0.003 ppm and 0.019 ± 0.001 ppm, respectively. Therefore, there is a drop in FWHM by ca. 35% for W36_U_ and 50% for W36_N_ at 1.1 GHz, relative to 600 MHz. In the aromatic region, significant line-narrowing is also observed at 1.1 GHz (Figure 4c,d). The two chosen resonances for this comparison are W36_U_ (ε3) and W36_U_ (*η*2). The FWHM values at 600 MHz are 0.034 ± 0.002 ppm and 0.026 ± 0.0002 ppm, respectively. At 1.1 GHz, the FWHM values decrease to 0.016 ± 5×10^−5^ ppm and 0.014 ± 0.0007 ppm, respectively. Hence, there is a decrease in FWHM by ca. 52% for W36_U_ (ε3) and 46% for W36_U_ (*η*2) at 1.1 GHz, relative to 600 MHz. The above results highlight that performing LC-photo-CIDNP at ultra-high field offers significant advantages for studying complex biomolecular systems, by combining the sensitivity advantage offered by nuclear-spin hyperpolarization with the improved spectral resolution achievable at 1.1 GHz.

**FIGURE 5 |:**
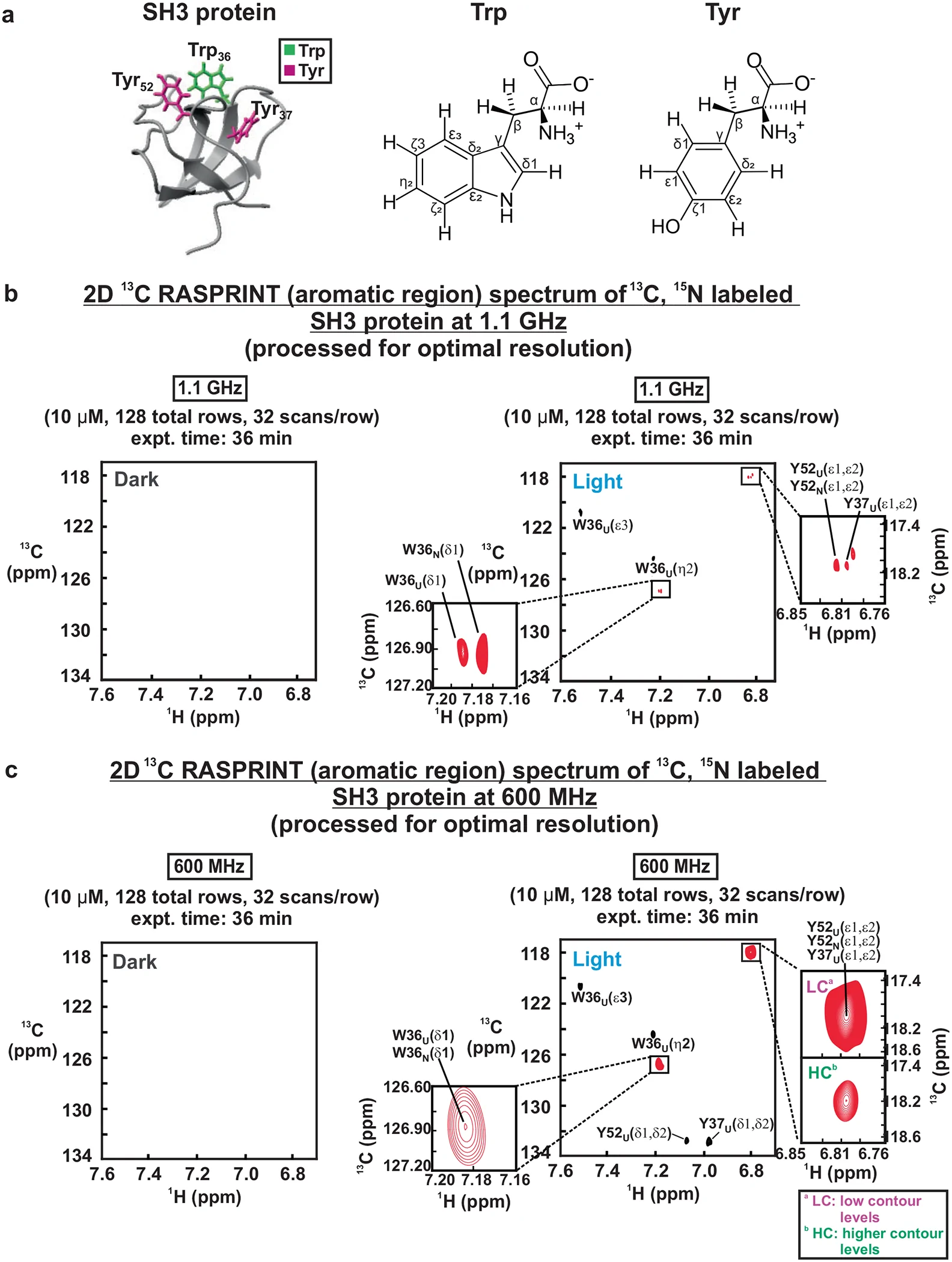
The 2D ^13^C RASPRINT spectrum of the SH3 protein displays a dramatic increase in sensitivity and resolution at 1.1 GHz. (a) Structure of native SH3 protein showing the solvent accessible LC-photo-CIDNP-active Trp and Tyr residues. (b) 2D ^13^C RASPRINT spectra (aromatic region) of ^13^C, ^15^N-labeled SH3 protein (10 *μ*M) at 1.1 GHz under dark (LED-off, left panel) and light (LED-on, right panel) conditions. The spectra at 1.1 GHz were acquired at 25°C with a sweep width of 16,666 Hz and 4096 total points in the direct dimension, and a sweep width of 8303 Hz, 128 total rows in the indirect dimension with 32 scans/row. (c) 2D ^13^C RASPRINT spectrum (aromatic region) of ^13^C,^15^N-labeled SH3 protein at 600 MHz (10 *μ*M) under dark (LED-off, left panel) and light (LED-on, right panel) conditions. The spectra at 600 MHz were collected at 25°C with a sweep width of 10,000 Hz and 4096 total points in the direct dimension and a sweep width of 6033 Hz and 128 total rows in the indirect dimension, with 32 scans/row. Black and red contours denote positive and negative resonances, respectively. A few resonances are shown as magnified insets. All spectra were processed identically for optimal resolution (see Section 3). The LED irradiation time and recycle delay were set to 0.3 s and 0.05 s, respectively. Dark spectra have identical vertical-scale and contour-level settings as their respective light spectra. All samples contain the fluorescein photosensitizer at 8 *μ*M concentration. See Section 3 for additional details. All data shown in this figure were collected on a 1.1 GHz spectrometer equipped with a cryogenic probe from Bruker (cryoprobe).

Next, we investigated ^13^C-^1^H correlations via 2D ^13^C RASPRINT to more accurately assess sensitivity and resolution tradeoffs at ultra-high field. Towards this end, we compared LC-photo-CIDNP hyperpolarization 2D data at 600 MHz and 1.1 GHz. The sensitivity advantage provided by LC-photo-CIDNP can be readily appraised by the fact that light spectra at both 600 MHz and 1.1 GHz show good S/N upon fast (36 min) 2D data collection on 10 μM SH3, while no signal is detected under dark conditions (Figure 5). On the other hand, the S/N values of the light spectra collected at 600 MHz and 1.1 GHz are comparable. This result shows that LC-photo-CIDNP data collection at 1.1 GHz is not particularly beneficial relative to 600 MHz from the NMR-sensitivity standpoint (Figure 5). Finally, two absorptive SH3 resonances Y52_U_ (δ1, δ2) and Y37_U_ (δ1, δ2) are visible at 600 MHz (Figure 5c) but are undetectable at 1.1 GHz under light conditions (Figure 5b). These resonances can, however, be detected at 1.1 GHz when the spectrum is processed with a different window function that optimizes sensitivity (Figure S2). The LC-photo-CIDNP-hyperpolarization spectral outcomes at 1.1 GHz and 600 MHz under light conditions show that ultra-high field does not provide any sensitivity advantage. These results are consistent with the theoretical predictions of Figure 3a.

While the 2D hyperpolarization spectra of 10 μM SH3 at 1.1 GHz do not show any improvement in S/N relative to 600 MHz, the ultra-high-field hyperpolarization bears some peculiar complementary benefits. Namely, a dramatic improvement in resolution is observed in highly crowded spectral regions, as highlighted in the insets of Figure 5b (at 1.1 GHz), relative to the corresponding insets of Figure 5c (at 600 MHz). For instance, the resonances centered at 118 ppm $\left(\delta_{13_{C}}\right)$, 6.8 ppm $\left(\delta_{1_{H}}\right)$ are clearly resolved at 1.1 GHz, as opposed to 600 MHz (Figure 5c). These resonances correspond to the aromatic ^1^H/^13^C Tyr nuclei of unfolded Y52_U_ (ε1, ε2), Y37_U_ (ε1, ε2) and native Y52_N_ (ε1, ε2), along with an additional presently unassigned resonance. Similarly, dramatic resolution improvements are also observed for the W36_U_ (δ1) and W36_N_ (δ1) 2D resonances at 1.1 GHz, relative to the data collected at 600 MHz. All spectral assignments were made based on known side-chain assignments deduced from 3D experiments [54].

### 2.5 | Hunting for LC-Photo-CIDNP Sensitivity Limits of 1.1 GHz: 100 nM QISP Trp Is Readily Detected

**FIGURE 6 |:**
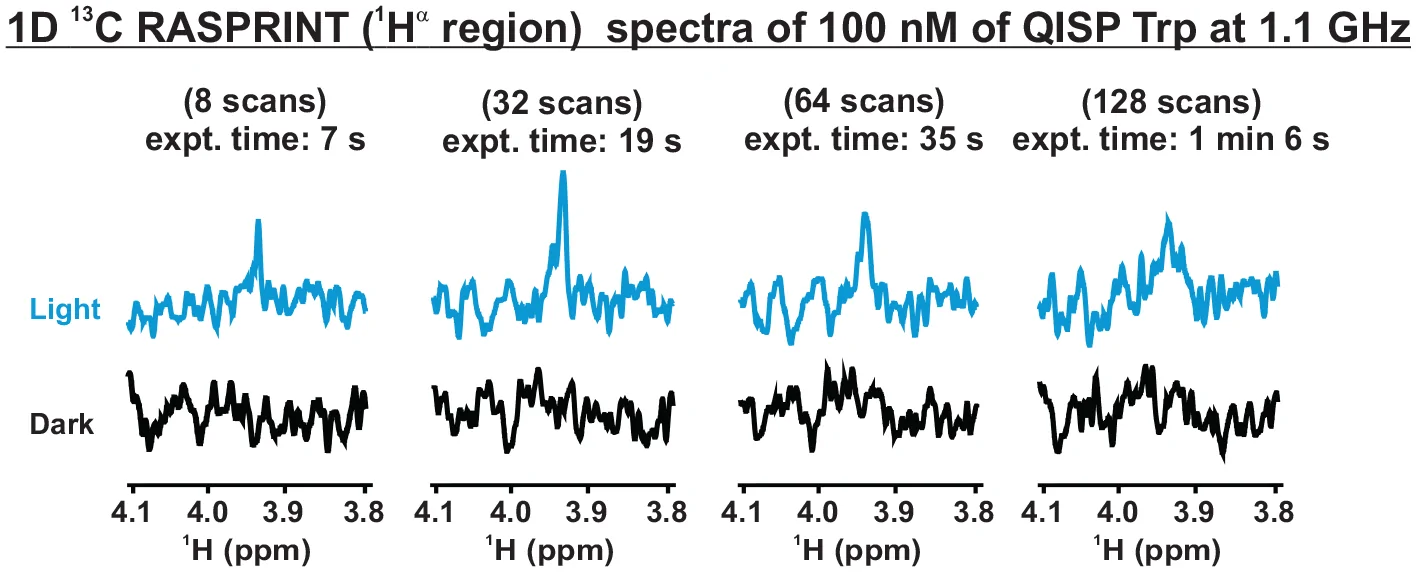
One-dimensional ^13^C RASPRINT spectra of highly diluted (100 nM) QISP Trp at 1.1 GHz. ^13^C RASPRINT (^1^H^α^ region) data were collected at an increasing number of scans at 25°C, with a sweep width of 12,500 Hz and 4096 total points. The LED irradiation time per scan and the recycle delay were 0.2 s and 0.05 s, respectively. The sample contained 2.5 *μ*M fluorescein photosensitizer. Even at a QISP-Trp concentration as low as 100 nM, the ^1^H^α^ resonance is observed within a few scans, demonstrating the high sensitivity achievable via LC-photo-CIDNP at ultra-high magnetic field. The 1.1 GHz spectrometer was equipped with a cryogenic probe from Bruker (cryoprobe).

In order to explore the sensitivity limits of hyperpolarization at ultra-high field, we performed LC-photo-CIDNP experiments on QISP Trp at a sub-micromolar concentration. Figure 6 shows a series of 1D ^1^H^α^-^13^C^α^ RASPRINT spectra of 100 nM QISP Trp at an increasing number of scans. The S/N initially increases up to 32 scans, consistent with expectations from signal averaging in the presence of hyperpolarization. On the other hand, accumulation of further transients (beyond 32 scans) leads to a decrease in S/N.

The above spectral behavior is consistent with the progressive photodegradation of target molecule and photosensitizer, which is known to occur for Trp upon prolonged data collection [28]. Interestingly, the extent of photodegradation is much reduced in the case of the SH3 protein, consistent with prior findings [28]. The enhanced SH3 photostability relative to its free aromatic amino acids is likely due to the slower translational diffusion of the protein and to the reduced solvent accessibility of its Trp/Tyr residues. These effects mitigate collision frequencies and overall exposure of SH3 to any photogenerated radicals that are responsible for biomolecular degradation [28]. In all, these features likely lead to the excellent spectral performance of SH3 in 2D experiments, even after more than 4000 scans (i.e., 32 × 128 = 4096), as shown in the previous section and in Figure 5.

## 3 | Experimental Section

### 3.1 | Materials

Fluorescein (sodium salt), vitamin C (ascorbic acid, VC), and the oxygen-scavenging enzymes glucose oxidase from *Aspergillus niger* (GO, EC 1.1.3.4, freeze-dried powder) and catalase from bovine liver (CAT, EC 1.11.1.6, freeze-dried powder) were purchased from MilliporeSigma. Uniformly ^13^C, ^15^N-labeled tryptophan (Trp-U-^13^C, ^15^N) was obtained from Cambridge Isotope Laboratories Inc. The selectively labeled Trp isotopolog Trp-α-^13^C-β,β,2,4,5,6,7-d_7_ (IUPAC name: (S)-2-amino-3-(2,4,5,6,7-^2^H_5_)-3-indolylpropionic acid), including a quasi-isolated spin pair (QISP) and denoted here as QISP Trp, was prepared and purified as described [25, 55]. Briefly, we performed an enzyme cascade reaction in the presence of formaldehyde-d_2_ (Cambridge Isotope Laboratories Inc.), glycine(2-^13^C) (Cambridge Isotope Laboratories Inc.), indole-d_7_ (CDN Isotopes), pyridoxal 5′-monophosphate (MilliporeSigma) and catalytic amounts of the *Pf* TrpB^2B9^ and *Tm* LTA enzymes. Identity of the purified QISP Trp was confirmed via ^1^H and ^13^C-NMR as described [25, 55]. The fluorescein photosensitizer was purchased as sodium salt (MilliporeSigma).

### 3.2 | SH3 Protein Preparation

The uniformly ^13^C, ^15^N-labeled SH3 protein was prepared as described [52]. In short, protein overexpression was carried out in *E. coli* BL21(DE3) cells in M9 minimal medium supplemented with ^15^N and ^13^C sources. Cells were cultured at 37°C to 0.6 optical density at 600 nm (OD_600_), followed by induction with isopropyl-β-D-1-thiogalactopyranoside (IPTG). After further growth to OD_600_ ≥ 1.2 and harvesting, cells were lysed, and the target protein was purified by anion-exchange chromatography followed by size-exclusion chromatography. Dialysis into aqueous buffer (50 mM Tris, 5 mM MgCl_2_, 50 mM KCl, pH 7.2) was then carried out, followed by aliquoting, flash-freezing in liquid nitrogen, and storage at −80°C. SH3 protein concentration was assessed by UV electronic absorption spectroscopy, using an extinction coefficient of 8480 M^−1^ cm^−1^ at 280 nm.

### 3.3 | LC-Photo-CIDNP NMR Sample Preparation

All LC-photo-CIDNP NMR samples (Trp-U-^13^C,^15^N, QISP Trp and ^13^C, ^15^N labeled SH3) were prepared as described [23, 27–29, 41], in 5% D_2_O containing 10 mM potassium phosphate buffer (pH 7.2), 0.15 μM glucose oxidase (GO), 0.10 μM catalase (CAT), 2.5 μM fluorescein and 2 μM vitamin C. In addition, D-Glucose (2.5 mM) was added ca. 10 min prior to data collection. Stock solutions of the GO and CAT oxygen-scavenging enzymes, fluorescein and the protein stock solutions were freshly thawed on ice before use. Vitamin C solutions were freshly prepared on the same day as the experiments. NMR measurements were carried out in 3 mm NMR tubes at 1.1 GHz and 5 mm NMR tubes at 600 MHz. DSS-d_6_ in D_2_O was used as an external chemical-shift reference for all 1D and 2D ^13^C RASPRINT experiments.

### 3.4 | LC-Photo-CIDNP Data Collection Under Dark Conditions for Enhancement-Factor Determination

In order to establish numerical values for the enhancement factors (ε), LC-photo-CIDNP data collection was carried out under dark conditions with high-concentration samples (see concentrations below) in 10 mM phosphate buffer (pH 7.2). Specifically, 100 μM QISP Trp and 5 mM Trp-U-^13^C,^15^N samples were used at 600 MHz, and 1.4 mM QISP Trp and 1.4 mM Trp-U-^13^C,^15^N samples were used at 1.1 GHz.

### 3.5 | LED Sources and Optical Irradiation Setup

The LED optical irradiation setup was as described [24]. Briefly, a UHP-FB-LED 450 light source (Prizmatix, Holon, Israel; emission frequency centered at 453 nm, Figure S3) was coupled to a polymer optical fiber with a 1.5 mm and 1.0 mm (POF; numerical aperture = 0.5, Prizmatix) in the case of data collection at 600 MHz and 1.1 GHz experiments, respectively [24]. Optical-fiber lengths of 4.1 m and 10 m were used in experiments at 600 MHz and 1.1 GHz, respectively. The output power measured at the fiber tip was 310 mW (600 MHz) and 300 mW (1.1 GHz). LED irradiation times are listed in pertinent figure legends. In the case of the 600 MHz experiments, the 4.1 m optical fiber (1.5 mm diameter) was inserted into a 4 mm NMR tube (427-PP-7, Wilmad-Labglass, Buena, NJ). The latter was then placed inside a 5 mm NMR tube in a coaxial configuration to ensure proper alignment of the irradiation within the sample volume. In the case of the 1.1 GHz experiments, the optical fiber (10 m, 1.0 mm diameter) was directly inserted into a 3 mm NMR tube containing the molecule of interest.

### 3.6 | Dependence of ^13^C RASPRINT Constant-Time Evolution on Resonance Phase

The ^1^H-detected ^13^C RASPRINT pulse sequence employed in this work includes a constant-time evolution period (T) that suppresses homonuclear ^13^C–^13^C J-couplings [24, 30]. As can be shown via the product-operator formalism, when T = 1/J_CC_ the phase of ^13^C resonances is inverted for carbons coupled to an odd number of neighboring carbons, relative to ^13^C resonances coupled to an even number of carbons. In contrast, when T = 2/J_CC_, all resonances have the same phase. Previous studies showed that T = 2/J_CC_ provides improved spectral resolution relative to T = 1/J_CC_, despite a slight loss in sensitivity due to increased T_2_ relaxation [53, 56]. In this work, T = 2/J_CC_ was used for all ^13^C RASPRINT experiments in the aromatic region, where spectral congestion is greater, while T = 1/J_CC_ was used for experiments in the ^1^H^α^ region. The emissive phases of the SH3 aromatic resonances of W36_N_ (δ1) and W36_U_ (δ1) in the LC-photo-CIDNP experiments of Figures 4c and 5b,c can be rationalized upon considering the intrinsic LC-photo-CIDNP phase of the ^13^C (δ1) resonances as well as the adopted constant-time evolution of T = 2/J_CC_. Similar arguments apply to the Tyr (Y) aromatic resonances, except that these display opposite photo-CIDNP phase relative to the corresponding Trp (W) phases. The resonance-phase dependence on the ^13^C RASPRINT constant-time duration (either T = 1/J_CC_ or T = 2/J_CC_) is systematically demonstrated experimentally in Figure S4 and further discussed in the accompanying legend.

#### 3.6.1 | ^13^C^α^ T_1_ Measurements

^13^C^α^ T_1_ experiments were carried out on a 600 MHz NMR spectrometer equipped with a cryoprobe from Bruker and on a 1.1 GHz NMR spectrometer equipped with either a cryo-probe or a room-temperature (RT) probe. QISP Trp and Trp-U-^13^C, ^15^N (100 μM) in 10 mM phosphate buffer (pH 7.2) and 10% D_2_O were analyzed with a ^13^C RASPRINT-like inversion recovery pulse sequence, as described [42]. Experiments at 1.1 GHz were performed with a 15 s recycle delay, 12,500 Hz sweep width and a 0.16 s acquisition time. Inversion recovery delays of 1 ms, 50 ms, 100 ms, 250 ms, 500 ms, 600 ms, 800 ms, 900 ms, 1.5 s, 3 s, 5 s and 10 s were used with 24 transients acquired per inversion recovery delay at 25°C. Data were fit to the following equation,

(5) I(t)=I(∞)(1−2etT1),

where *I*(*t*) is the resonance intensity at any inversion-recovery delay time *t*, and *I* (∞) is the resonance intensity when the magnetization is at fully thermal equilibrium. The TopSpin 4.5.0 Dynamics T_1_/T_2_ module was used to fit the data at 600 MHz and 1.1 GHz. One set of T_1_ measurements was performed using a RT probe at 1.1 GHz. On the other hand, we do not expect the probe type to have any effect on T_1_ values. The ^13^C^α^ T_1_ results are summarized in Table S1. The ^13^C^α^ T_1_ values at 600 MHz are reported in the literature [42]. The published ^13^C^α^ T_1_ values of two Trp isotopologs at 600 MHz and our own data at 1.1 GHz are the same within error (*p* = 0.31 for Trp-U-^13^C,^15^N and *p* = 0.35 for QISP Trp).

#### 3.6.2 | ^1^H^α^ T_2_ Measurements

^1^H^α^ T_2_ relaxation time measurements were carried out on a 600 MHz spectrometer (with a cryoprobe from Bruker) and 1.1 GHz (with a RT probe) instruments. Either 1.4 mM QISP Trp or Trp-U-^13^C, ^15^N in 10 mM phosphate buffer (pH 7.2) and 10% D_2_O were used. T_2_ data were collected with a CPMG experiment including a double spin echo, as described [25, 26, 57]. Presaturation during recycle delay was used for solvent suppression, and ^13^C-decoupling (GARP) was used during acquisition. The results are shown in Table S2. A recycle delay of 5 s, a 13,889 Hz sweep width, and an acquisition time of 0.2 s were used at 1.1 GHz. A recycle delay of 5 s, a 12,019 Hz sweep width, and an acquisition time of 0.3 s were used at 600 MHz. The duration of each CPMG block was 50 ms, with each individual CPMG delays set to 12.5 ms. A total of 80 CPMG blocks were used, corresponding to total CPMG time of 4 s to ensure complete ^1^H^α^ transverse relaxation. Data were fit to

(6) $$M(t)=lnM(0)-\left(\frac{1}{T_{2}}\right)\;t,$$

where *M*(*t*) denotes the resonance area of ^1^H^α^, t is the total CPMG time [25]. The QISP Trp isotopolog shows longer *T*_2_ values than Trp-U-^13^C, ^15^N. However, ^1^H^α^ *T*_2_ values of each of the two Trp isotopologs at 600 MHz and 1.1 GHz are the same within error (*p* = 0.31 for QISP Trp and *p* = 0.68 for Trp-U-^13^C,^15^N).

### 3.7 | LC-Photo-CIDNP NMR Experiments at 600 MHz and 1.1 GHz

All LC-photo-CIDNP experiments were performed with a UHP-FB-LED 450 optical irradiation source, which was delivered into the NMR tubes through a polymeric optical fiber (see details in a previous section). NMR data at 600 MHz were collected on a Bruker spectrometer (Avance III console) equipped with a ^1^H{^19^F/^13^C/^15^N} triple-resonance (TCI-F) cryogenic probe fitted with z-gradients. Data at 1.1 GHz were collected on a Bruker spectrometer (AV NEO console) equipped with either a ^1^H{^13^C/^15^N} CP2.1 cryogenic probe fitted with *z*-gradients, or with a TXI H/C/N/D 3 mm room-temperature probe with z-axis gradients. Carrier frequencies of ^13^C RASPRINT experiments were centered at 4.70 ppm (^1^H channel), and at 55 ppm and 120 ppm (^13^C channel) for the C^α^ and C^aromatic^ regions, respectively. 1D, ^13^C RASPRINT under light and dark conditions employed 4096 total points, an acquisition time of 0.1638 s and 0.243 s respectively, a recycle delay of 0.05 s (unless otherwise mentioned) and an irradiation time of 0.2 s. The 1D ^13^C RASPRINT data collected on high-concentration samples (for ε determinations) employed long recycle delays (5 s at 600 MHz and 10 s at 1.1 GHz) to prevent ε overestimations from incomplete longitudinal relaxation between scans [25, 26]. The full-width-at-half-maximum (FWHM, in ppm or Hz) data in Figure 4 were determined within TopSpin 4.5.0 with the “peakw” command. The 2D ^13^C RASPRINT spectra were acquired with 4096 total points in the direct dimension and either 64 or 128 total points in the indirect dimension. All 2D ^13^C RASPRINT experiments at 1.1 GHz were collected with a sweep width of 16,666 Hz (15.1 ppm) in the direct dimension and 8303 Hz (30 ppm) in the indirect dimension. All 2D NMR data at 600 MHz were collected with a sweep width of 10,000 Hz (16.7 ppm) in the direct dimension and 6033 Hz (40 ppm) in the indirect dimension. All 1D and 2D ^13^C RASPRINT experiments included 4 dummy scans lacking LED irradiation as described [24]. The 1D ^13^C RASPRINT data were processed with MestReNova (version 15.1.0) upon apodization with a 5 Hz exponential line-broadening function and zero-filling to 65, 536 complex points. All 2D spectra were processed with NMRPipe (v. 9.0.0-b10) and visualized with NMRDraw and NMRViewJ (v. 2009.015.15.35) [58, 59]. In both 600 MHz and 1.1 GHz experiments, the direct dimension was zero-filled to 8192 complex points while the indirect dimension was zero-filled to 2048 complex points. All spectra in Figure 5 were processed for optimal resolution using a 10% shifted Gaussian window function in both dimensions. The spectrum shown in Figure S2 was processed for optimal sensitivity with an unshifted cosine-bell-square apodization in direct and indirect dimensions. Known NMR assignments for Trp [30] and backbone and side chains of SH3 [51, 54] were used.

## 4 | Conclusions

In summary, this study shows that LC-photo-CIDNP hyperpolarization can be achieved in liquids at both 14.1 T (600 MHz) and 25.9 T (1.1 GHz). Hence, this NMR sensitivity-enhancement technology performs well in liquids across a wide range of magnetic fields. In agreement with theoretical predictions, LC-photo-CIDNP of Trp employing fluorescein as a photosensitier exhibits a lower degree of hyperpolarization at 1.1 GHz than at 600 MHz. On the other hand, LC-photo-CIDNP hyperpolarization at ultra-high field (1.1 GHz) is a remarkably useful and powerful tool to further increase NMR sensitivity relative to conventional NMR spectroscopy. This goal is achieved by providing up to 160-fold enhancement factors (ε), relative to “dark” hyperpolarization-free experiments. Most importantly, hyperpolarization within the ultra-high-field environment offers unprecedented advantages in terms of combining high sensitivity and high resolution. This concept is particularly important in the case of proteins. For instance, significantly sharper lineshapes that were achieved in solution for the hyperpolarized 10 μM SH3-protein at 1.1 GHz (in both 1D and 2D spectra), relative to data collected at 600 MHz. In all, LC-photo-CIDNP at ultra-high *B*_0_ offers a unique avenue to probe biomolecular structure in solution at an unprecedented degree of concurrently high sensitivity and resolution.

## Supplementary Material

Supplementary Materials

Additional supporting information can be found online in the Supporting Information section. **Table S1:** jccs70254-sup-0001-TableS1-S2-FigureS1-S4.pdf. ^13^C^*α*^ nuclear T_1_ values of Trp-U-^13^C, ^15^N and QISP Trp at 600 MHz and 1.1 GHz. **Table S2:** ^1^H^α^ T_2_ values of Trp-U-^13^C,^15^N and QISP Trp at 600 MHz and 1.1 GHz. **Figure S1:** Comparison between full width at half maximum (FWHM, in Hz) values of folded and unfolded states of the SH3 protein at 600 MHz and 1.1 GHz in the presence of LC-photo-CIDNP hyperpolarization. This bar graph illustrates the full-width-at-half-maximum (FWHM, in Hz) of the W36N and W36U resonances of the SH3 protein. Data were collected with the 13C RASPRINT (1Hα region) pulse sequence under light (LED-on) conditions (*n* = 2, at each field). FWHM values (in Hz) of the W36N and W36U SH3 resonances at 1.1 GHz are 36.5 ± 3.4 and 21.2 ± 1.0, respectively. At 600 MHz, FWHM values (in Hz) of the W36N and W36U SH3 resonances are 30.5 ± 1.3 and 22.8 ± 1.9, respectively. Spectra were acquired on 10 μM SH3 samples at 25°C using a spectral width of 16,666 Hz (1.1 GHz) or 10,000 Hz (600 MHz), with 4096 complex points and 256 scans. The recycle delay was 0.05 s, and the LED irradiation time was 0.2 s per scan. **Figure S2:** 2D 13C RASPRINT spectra of 13C, 15N labeled SH3 protein at 1.1 GHz. 2D 13C RASPRINT spectra acquired under dark (LED-off, left panel) and light (LED-on, right panel) conditions. Black and red contours denote positive and negative resonances, respectively. The LED irradiation time per scan and recycle delay were 0.3 s and 0.05 s, respectively. The sample contained 8 mM of the fluorescein photosensitizer. The spectra at 1.1 GHz were acquired at 25°C with a sweep width of 16,666 Hz and 4096 total points in the direct dimension, and a sweep width of 8303 Hz and 128 total rows in the indirect dimension, with 32 scans/row. See Section 3 for details. Data were processed for optimal sensitivity with an unshifted cosine-bell-square window function in the direct and indirect dimensions, respectively. **Figure S3:** Emission spectrum of the LED employed in this work. The UHP-mic-450 nm LED (Prizmatix) emission spectrum shown above was acquired with an HR2 high-resolution spectrophotometer (OceanOptics). Data are displayed as spectral power density (i.e., power per unit wavelength). The emission-band intensities are normalized relative to the maximum at λmax = 453 nm. **Figure S4:** Dependence of constant-time evolution (T) on resonance phase in 13C RASPRINT. (a) 1D 1H-detected 13C RASPRINT spectra of Trp-U-13C, 15N acquired at high concentration (5 mM) under LC-photo-CIDNP dark conditions (i.e., LED-off). The spectra show a negative phase for the δ1 resonance and positive phases for all other resonances, when the constant time setting is T = 1/JCC. In contrast, when T = 2/JCC is used, the δ1 resonance acquires a positive phase. As a reference, a standard 1D 13C NMR pulse-acquire spectrum of Trp-U-13C, 15N, including 1H decoupling during acquisition, is also provided on the left-hand side of the panel. (b) 1H-detected 1D 13C RASPRINT and 1D 13C LC-photo-CIDNP spectra of Trp-U-13C, 15N acquired at 100 μM concentration under LC-photo-CIDNP light (i.e., LED-on) and dark (i.e., LED off) conditions. The 1D 13C LC-photo-CIDNP experiment includes a simple optical irradiation-pulse-acquire scheme and is designed to visualize the effect of the photo-CIDNP-related resonance-phase alone, in the absence of the constant-time evolution which further affects resonance phases, and which is present in the 13C RASPRINT pulse sequence. Under light conditions, the 13C RASPRINT spectrum acquired with T = 1/JCC displays a δ1 resonance with identical phase relative to the other resonances (ε3 and η2), as opposed to the reference 13C LC-photo-CIDNP experiment (on the left-hand side of the panel), where the δ1 resonance has an opposite phase relative to ε3 and η2. In contrast, when T = 2/JCC is used in 13C RASPRINT, the δ1 resonance acquires an opposite phase relative to the other resonances, as shown in the 13C RASPRINT spectrum on the right-hand-side. All of the observed resonance phases are fully accounted for based on the experimental data and conceptual arguments presented here and in the main manuscript. The 100 μM LC-photo-CIDNP sample of Trp-U-13C, 15N contained 25 μM of the fluorescein photosensitizer. See methods for details. Note that all the 13C RASPRINT aromatic-region spectra of the SH3 protein presented in the main manuscript (Figures 4c and 5b,c) were acquired with T = 2/JCC. This constant-time setting preserves the relative resonance phases established by 13C photo-CIDNP. Note that the optical irradiation that generates 13C photo-CIDNP precedes the constant-time evolution T, in the 13C RASPRINT pulse sequence.

## Figures and Tables

**TABLE 1 |:** Experimental LC-photo-CIDNP enhancement factors (ε), percent polarizations (P_%_) and sensitivities (Sens_*C*_) displayed by Trp-U-^13^C,^15^N and QISP Trp at either 1.1 GHz or 600 MHz.

	Enhancement factor (ε)^b^		Percent polarization (P_%_)^c^		LC-photo-CIDNP sensitivity (Sens_*c*_)^d^		
Trp isotopolog^a^	1.1 GHz (RT-probe)^e^	600 MHz (cryo-probe)	1.1 GHz (RT-probe)^e^	600 MHz (cryo-probe)	1.1 GHz (RT—probe)^e^	1.1 GHz (cryo-probe)	600 MHz (cryo-probe)
QISP Trp	104.8 ± 18.8	487.6 ± 18.5	0.2 ± 0.04	0.6 ± 0.02	5.2 ± 0.38	29.1 ± 3.7	108.9 ± 2.3
Trp-U-13C, 15N	160.1 ± 16.0	261.2 ± 68.3	0.4 ± 0.04	0.3 ± 0.08	2.6 ± 0.16	13.2 ± 2.4	18.5 ± 1.7

a All data were collected at 25°C with the 1D ^13^C RASPRINT pulse sequence (^1^H^α^ region). Trp-U-^13^C, ^15^N was used as a reference compound. Unless otherwise stated, all calculations used LC-photo-CIDNP data (collected under light conditions) from a 2.5 μM sample with 32 scans, a 0.05 s recycle delay, and 0.2 s LED irradiation per scan. Data are shown as average ± standard error for *n* = 2, at 600 MHz (with cryogenic probe), 1.1 GHz (with a RT probe) and 1.1 GHz (with a cryogenic probe). See Section 3 for details.

b Determined from data in Figure 3c.

c Determined from data in Figure 3d.

d Determined from data in Figure 3b. Sens_*C*_ values were calculated via the “.sino” command (TopSpin 4.5.0) and Equation (1).

e RT probe denotes a room-temperature probe.

**TABLE 2 |:** Quantitative evaluation of light and dark contributions to the sensitivity (Sens_*C*_) of LC-photo-CIDNP experiments performed on different Trp isotopologs at 600 MHz.

Trp isotopolog^a^	LC-photo-CIDNP hyperpolarization effect (light effect)^b^	Linewidth reduction (dark effect)^c^	Elimination of coherence losses during pulse sequence (dark effect)^d^	Product of individual contributions	Overall LC-photo-CIDNP sensitivity (Sens_*C*_)
Trp-U-^13^C, ^15^N	100.0	100.0	100.00	100.00	100.00
QISP Trp	198.4 ± 44.82	177.0 ± 3.5	142.4 ± 21.0	500.1 ± 14.7	590.1 ± 68.0

a All data were collected with the 1D ^13^C RASPRINT pulse sequence (^1^H^α^ region). Trp-U-^13^C,^15^N was used as a reference compound. Data shown here were collected on a 600 MHz spectrometer equipped with a cryogenic probe.

b Determined from data in Figure 3d.

c Determined from ^1^H^α^ T_2_ values under dark conditions for 1.4 mM Trp-U-^13^C,^15^N and 1.4 mM QISP Trp samples in 10 mM phosphate buffer (pH ~7.2), with *n* = 3 for QISP Trp and *n* = 2 for Trp-U-^13^C,^15^N at 600 MHz (see Section 3 for details).

d Determined from resonance-area values under dark conditions in 10 mM phosphate buffer at pH 7.2 (*n* = 2).

**TABLE 3 |:** Quantitative evaluation of light and dark contributions to the sensitivity (Sens_*C*_) of LC-photo-CIDNP experiments performed on different Trp isotopologs at 1.1 GHz.

Trp isotopolog^a^	LC-photo-CIDNP hyperpolarization effect (light effect)^b^	Linewidth reduction (dark effect)^c^	Elimination of coherence losses during pulse sequence (dark effect)^d^	Product of individual contributions	Overall LC-photo-CIDNP sensitivity (Sens_*C*_)
Trp-U-^13^C, ^15^N	100.0	100.0	100.00	100.00	100.00
QISP Trp	65.0 ± 5.2	166.4 ± 4.0	206.4 ± 32.0	223.2 ± 11.2	196.7 ± 1.9

a All data were collected with the 1D ^13^C RASPRINT pulse sequence (^1^H^α^ region). Trp-U-^13^C,^15^N was used as a reference compound. Data shown here were collected on a 1.1 GHz spectrometer equipped with a room-temperature (RT) probe.

b Determined from data in Figure 3d.

c Determined from 1H^α^ T_2_ values under dark conditions for 1.4 mM Trp-U-^13^C,^15^N and 1.4 mM QISP Trp samples in 10 mM phosphate buffer (pH ~7.2) with *n* = 3 for QISP Trp and *n* = 2 for Trp-U-^13^C, ^15^N at 1.1 GHz (see Section 3 for details).

d Determined from the resonance-area values under the curve under dark conditions in 10 mM phosphate buffer at pH 7.2 (*n* = 2).

## Data Availability

The data that support the findings of this study are available from the corresponding author upon reasonable request.
