# Supplementary material for: Nuclear-Spin Hyperpolarization of Biomolecules by Optically Enhanced NMR at Ultra-High Field
Source: J Chin Chem Soc. Author manuscript; Available in PMC 2026 Aug 19. (PMC13485145; doi:10.1002/jccs.70254)
Supplement: Supplementary file 1 [file NIHMS2202573-supplement-Supplementary_Materials.pdf]

Supplementary Materials

Nuclear-Spin Hyperpolarization of Biomolecules by Optically Enhanced NMR at Ultra-High Field

Ji Ho Jeong[§], Anubhab Halder[§], Silvia Cavagnero*

Department of Chemistry, University of Wisconsin-Madison, 1101 University Ave, Madison, WI, 53706

* Corresponding author (cavagnero@chem.wisc.edu)

[§] Equal contributors

Keywords: NMR; photo-CIDNP; LC-photo-CIDNP; hyperpolarization; high field; sensitivity.

Supplementary Tables

Table S1. $^{13}C^{\alpha}$ nuclear T_1 values of Trp-U-^{13}C, ^{15}N and QISP Trp at 600 MHz and 1.1 GHz.

Trp isotopolog	T_1 (s) [a]	
	**1.1 GHz**	**600 MHz**
**Trp-U-^{13}C, ^{15}N**	1.32 ± 0.02	1.35 ± 0.01
**QISP Trp**	1.37 ± 0.09	1.48 ± 0.02

[a] Each T_1 value is shown as average ± SE (n=2, at both fields). See Experimental Section for details.

Table S2. $^1H^{\alpha}$ T_2 values of Trp-U-^{13}C, ^{15}N and QISP Trp at 600 MHz and 1.1 GHz.

Trp isotopolog	T_2 (ms) [a]	
	**1.1 GHz**	**600 MHz**
**Trp-U-^{13}C, ^{15}N**	409.2 ± 25.2	397.2 ± 5.7
**QISP Trp**	680.9 ± 18.8	703.1 ± 8.2

[a] Each T_2 value is shown as average ± SE (n=3 for QISP Trp and n=2 for Trp-U-^{13}C, ^{15}N, at both fields). See Experimental Section for details.

Supplementary Figures

Figure S1

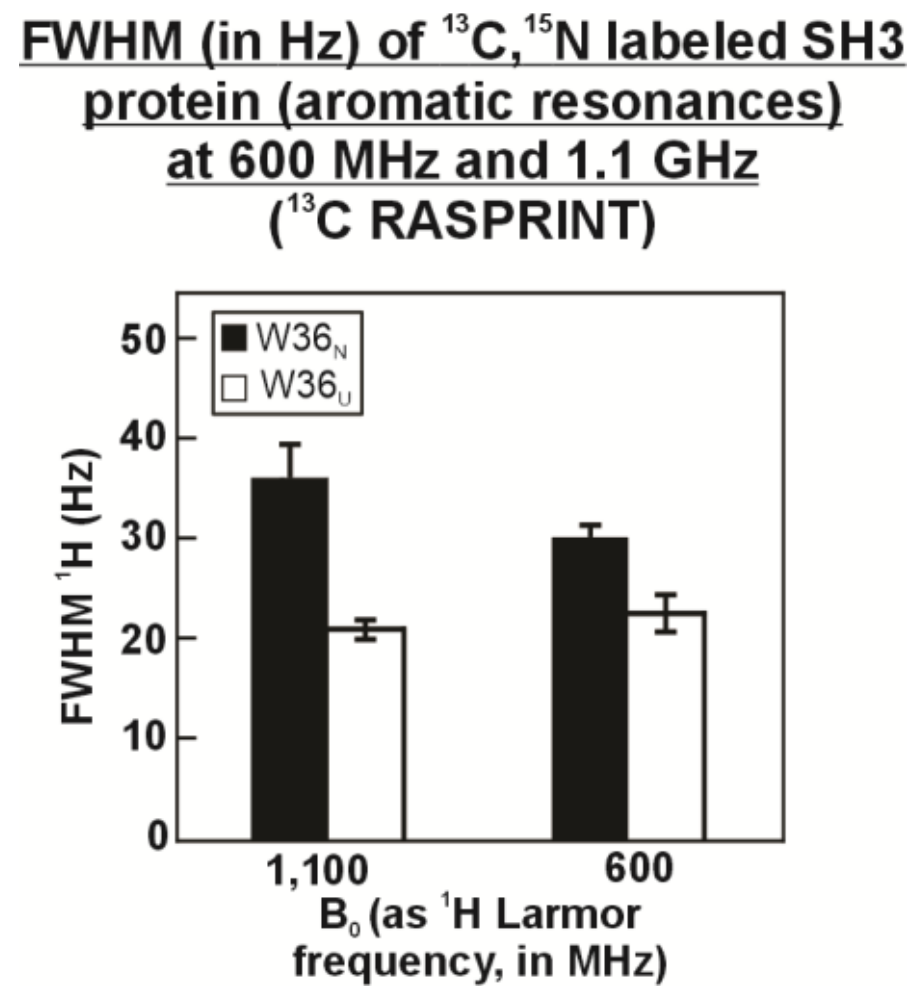

Figure S1. Comparison between full width at half maximum (FWHM, in Hz) values of folded and unfolded states of the SH3 protein at 600 MHz and 1.1 GHz in the presence of LC-photo-CIDNP hyperpolarization. This bar graph illustrates the full-width-at-half-maximum (FWHM, in Hz) of the $W36_N$ and $W36_U$ resonances of the SH3 protein. Data were collected with the ^{13}C RASPRINT ($^1H^\alpha$ region) pulse sequence under light (LED-on) conditions (n = 2, at each field). FWHM values (in Hz) of the $W36_N$ and $W36_U$ SH3 resonances at 1.1 GHz are 36.5 ± 3.4 and 21.2 ± 1.0, respectively. At 600 MHz, FWHM values (in Hz) of the $W36_N$ and $W36_U$ SH3 resonances are 30.5 ± 1.3 and 22.8 ± 1.9, respectively. Spectra were acquired on 10 µM SH3 samples at 25 °C using a spectral width of 16,666 Hz (1.1 GHz) or 10,000 Hz (600 MHz), with 4,096 complex points and 256 scans. The recycle delay was 0.05 s, and the LED irradiation time was 0.2 s per scan.

Figure S2

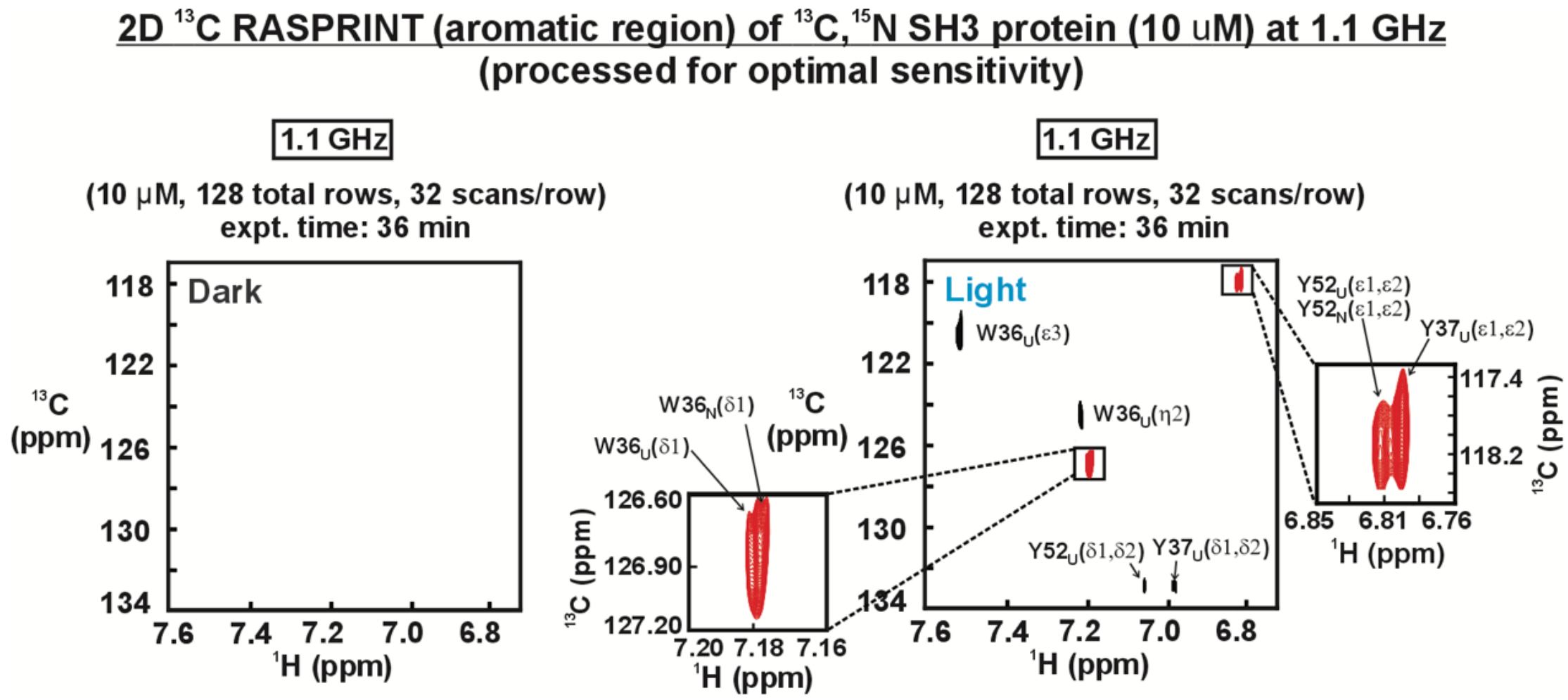

Figure S2. 2D ^{13}C RASPRINT spectra of ^{13}C, ^{15}N labeled SH3 protein at 1.1 GHz. 2D ^{13}C RASPRINT spectra acquired under dark (LED-off, left panel) and light (LED-on, right panel) conditions. Black and red contours denote positive and negative resonances, respectively. The LED irradiation time per scan and recycle delay were 0.3 s and 0.05 s, respectively. The sample contained 8 mM of the fluorescein photosensitizer. The spectra at 1.1 GHz were acquired at 25 ºC with a sweep width of 16,666 Hz and 4,096 total points in the direct dimension, and a sweep width of 8,303 Hz and 128 total rows in the indirect dimension, with 32 scans/row. See Experimental Section for details. Data were processed for optimal sensitivity with an unshifted cosine-bell-square window function in the direct and indirect dimensions, respectively.

Figure S3

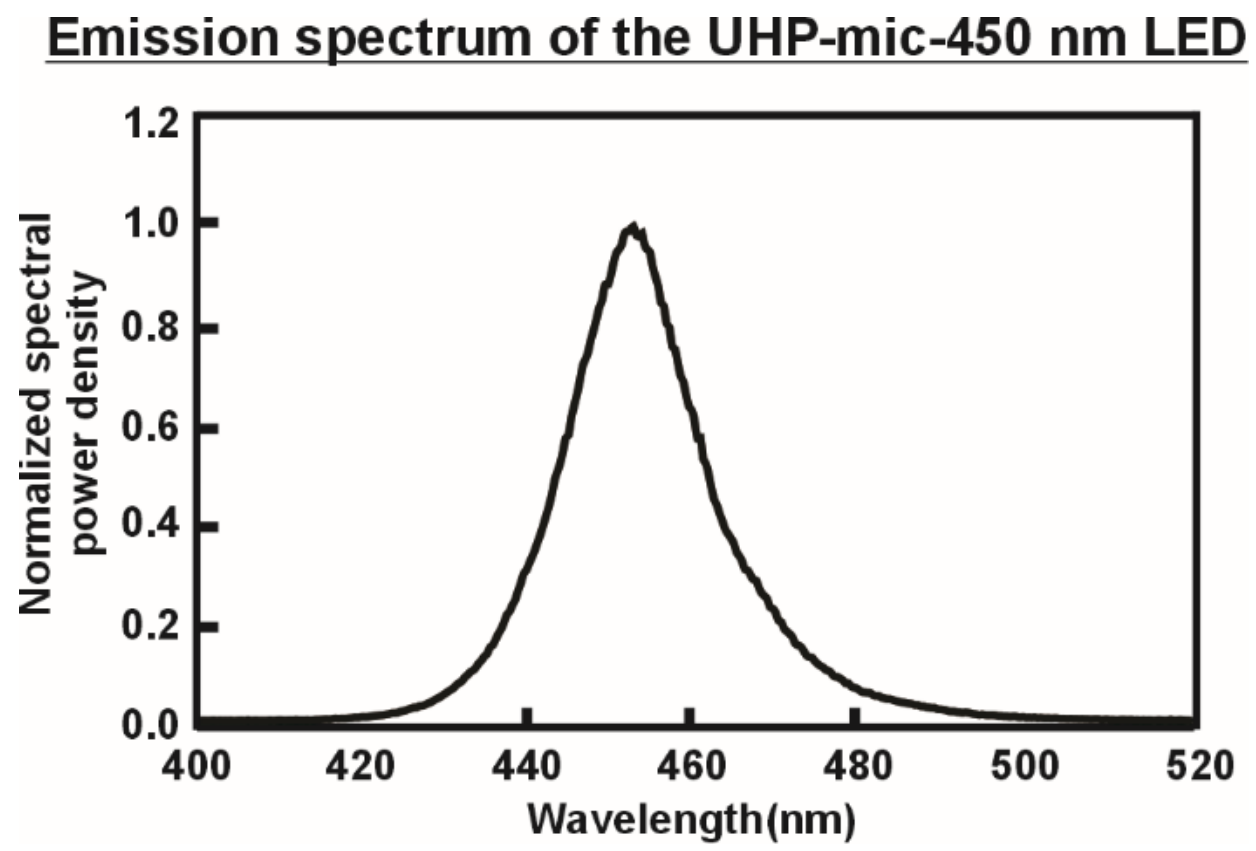

Figure S3. Emission spectrum of the LED employed in this work. The UHP-mic-450 nm LED (Prizmatix) emission spectrum shown above was acquired with an HR2 high-resolution spectrophotometer (OceanOptics). Data are displayed as spectral power density (i.e., power per unit wavelength). The emission-band intensities are normalized relative to the maximum at λ_{max} = 453 nm.

Figure S4

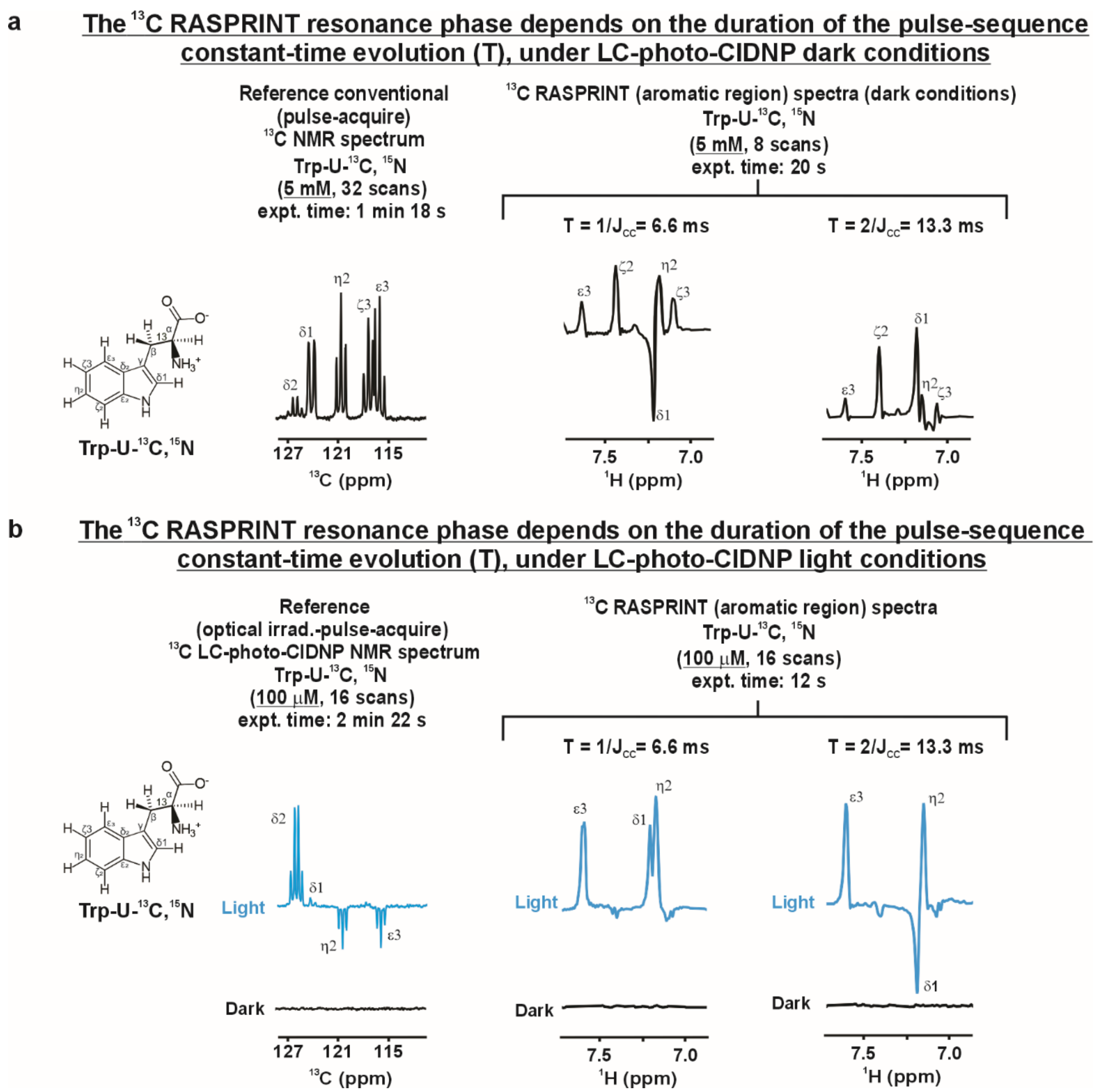

Figure S4. Dependence of constant-time evolution (T) on resonance phase in ^{13}C RASPRINT. (a) 1D ^{1}H-detected ^{13}C RASPRINT spectra of Trp-U-^{13}C, ^{15}N acquired at high concentration (5 mM) under LC-photo-CIDNP dark conditions (i.e., LED-off). The spectra show a negative phase for the δ_1 resonance and positive phases for all other resonances, when the constant time setting is T = 1/J$_{CC}$. In contrast, when T = 2/J$_{CC}$ is used, the δ_1 resonance acquires a positive phase. As a

reference, a standard 1D ^{13}C NMR pulse-acquire spectrum of Trp-U-^{13}C, ^{15}N, including ^{1}H decoupling during acquisition, is also provided on the left-hand side of the panel. (b) ^{1}H-detected 1D ^{13}C RASPRINT and 1D ^{13}C LC-photo-CIDNP spectra of Trp-U-^{13}C, ^{15}N acquired at 100 μM concentration under LC-photo-CIDNP light (i.e., LED-on) and dark (i.e., LED off) conditions. The 1D ^{13}C LC-photo-CIDNP experiment includes a simple optical irradiation-pulse-acquire scheme and is designed to visualize the effect of the photo-CIDNP-related resonance-phase alone, in the absence of the constant-time evolution which further affects resonance phases, and which is present in the ^{13}C RASPRINT pulse sequence. Under light conditions, the ^{13}C RASPRINT spectrum acquired with $T = 1/J_{CC}$ displays a δ_1 resonance with identical phase relative to the other resonances (ε_3 and η_2), as opposed to the reference ^{13}C LC-photo-CIDNP experiment (on the left-hand side of the panel), where the δ_1 resonance has an opposite phase relative to ε_3 and η_2. In contrast, when $T = 2/J_{CC}$ is used in ^{13}C RASPRINT, the δ_1 resonance acquires an opposite phase relative to the other resonances, as shown in the ^{13}C RASPRINT spectrum on the right-hand-side. All of the observed resonance phases are fully accounted for based on the experimental data and conceptual arguments presented here and in the main manuscript. The 100 μM LC-photo-CIDNP sample of Trp-U-^{13}C, ^{15}N contained 25 μM of the fluorescein photosensitizer**.** See methods for details. Note that all the ^{13}C RASPRINT aromatic-region spectra of the SH3 protein presented in the main manuscript (Figures 4c and 5b,c) were acquired with $T = 2/J_{CC}$. This constant-time setting preserves the relative resonance phases established by ^{13}C photo-CIDNP. Note that the optical irradiation that generates ^{13}C photo-CIDNP precedes the constant-time evolution T, in the ^{13}C RASPRINT pulse sequence.
